# Machine Learning-Assisted Identification and Quantification of Hydroxylated Metabolites of Polychlorinated Biphenyls in Animal Samples

**DOI:** 10.1021/acs.est.2c02027

**Published:** 2022-09-01

**Authors:** Chun-Yun Zhang, Xueshu Li, Kimberly P. Keil Stietz, Sunjay Sethi, Weizhu Yang, Rachel F. Marek, Xinxin Ding, Pamela J. Lein, Keri C. Hornbuckle, Hans-Joachim Lehmler

**Affiliations:** † Department of Occupational and Environmental Health, The University of Iowa, Iowa City, Iowa 52242, United States; ‡ Department of Molecular Biosciences, School of Veterinary Medicine, University of California Davis, Davis, California 95616, United States; § Department of Pharmacology and Toxicology, College of Pharmacy, University of Arizona, Tucson, Arizona 85721, United States; ∥ Department of Civil and Environmental Engineering and IIHR Hydroscience and Engineering, The University of Iowa, Iowa City, Iowa 52242, United States

**Keywords:** OH-PCBs, GC-MS/MS method, model prediction, relative retention time, relative response factor

## Abstract

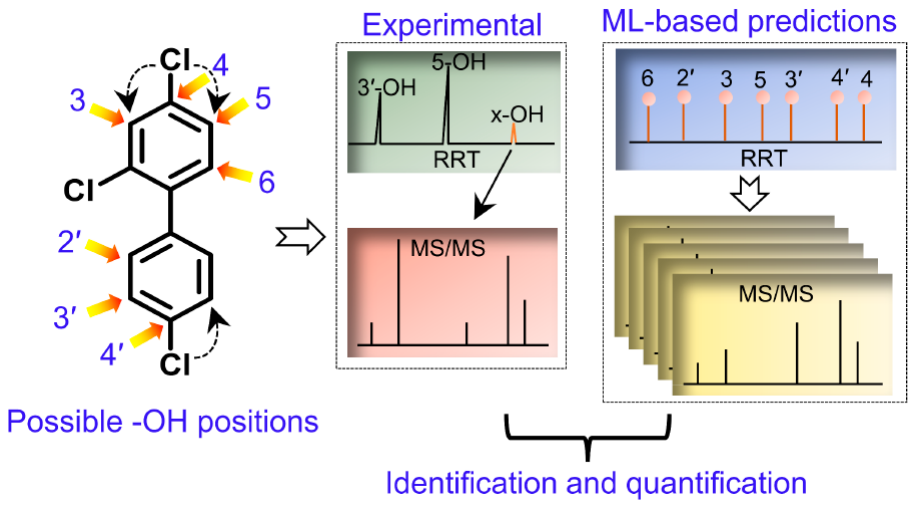

Laboratory studies of the disposition and toxicity of hydroxylated polychlorinated biphenyl (OH-PCB) metabolites are challenging because authentic analytical standards for most unknown OH-PCBs are not available. To assist with the characterization of these OH-PCBs (as methylated derivatives), we developed machine learning-based models with multiple linear regression (MLR) or random forest regression (RFR) to predict the relative retention times (RRT) and MS/MS responses of methoxylated (MeO-)PCBs on a gas chromatograph-tandem mass spectrometry system. The final MLR model estimated the retention times of MeO-PCBs with a mean absolute error of 0.55 min (*n* = 121). The similarity coefficients cos θ between the predicted (by RFR model) and experimental MS/MS data of MeO-PCBs were >0.95 for 92% of observations (*n* = 96). The levels of MeO-PCBs quantified with the predicted MS/MS response factors approximated the experimental values within a 2-fold difference for 85% of observations and 3-fold differences for all observations (*n* = 89). Subsequently, these model predictions were used to assist with the identification of OH-PCB 95 or OH-PCB 28 metabolites in mouse feces or liver by suggesting candidate ranking information for identifying the metabolite isomers. Thus, predicted retention and MS/MS response data can assist in identifying unknown OH-PCBs.

## Abstract 2

Machine learning-based models were used to identify and quantify toxicologically relevant hydroxylated PCB metabolites in biological samples.

## Introduction

Polychlorinated biphenyls (PCBs) are a class of environmental pollutants that can be transformed into hydroxylated PCBs (OH-PCBs) by reaction with hydroxyl radicals in the environment^1,2^ or via oxidation by cytochrome P450 enzymes in organisms.^3^ OH-PCBs are also present in technical PCB mixtures.^4^ A total of 837 monohydroxylated PCBs (mono-OH-PCBs) and thousands of dihydroxylated PCBs (di-OH-PCBs) can be formed from the 209 possible PCB congeners.^3^ The parent PCBs are still present in the environment, human diet, and humans^5−9^ and can be found in consumer products, such as paints and silicon rubber.^10−13^ Therefore, it is not surprising that many OH-PCB congeners have been detected in environmental or biological media.^4,14−16^ OH-PCBs are potentially more toxic than the corresponding parent PCBs.^3^ For example, OH-PCBs can interact with nuclear transcription factors, such as the aryl hydrocarbon receptor, constitutive androstane receptor, and pregnane X receptor.^17,18^ They are endocrine-disrupting chemicals that, for example, inhibit estrogen sulfotransferase and bind to transthyretin.^18−22^ Di-OH-PCBs are oxidation products of mono-OH-PCBs, with PCB catechols being central PCB metabolites in mammals.^23−25^ Di–OH-PCB metabolites can be transformed into PCB quinones, reactive PCB metabolites that cause oxidative stress or covalently bind to DNA and other cellular targets.^26−29^ Some PCB catechols are tumor initiators in the liver.^30,31^

Despite the well-documented toxicity of OH-PCBs, their presence in environmental samples, wildlife, laboratory animals, and humans has not been fully characterized, partly because of the lack of authentic analytical standards. OH-PCBs are typically analyzed as methylated derivatives (MeO-PCBs) with gas chromatographic (GC) methods.^23,32,33^ GC can also be used to identify and quantify other PCB metabolites, such as PCB sulfates, as MeO-PCBs after deconjugation and derivatization.^34^ GC coupled with tandem mass spectrometry (GC-MS/MS) is a useful method to quantify the MeO-PCBs because of its good separation, high selectivity, and low detection limits for this class of compounds.^4,14,15^ However, only a small number of the 837 possible OH-PCB congeners, either as hydroxylated or methoxylated derivatives,^35^ are available. The lack of analytical standards represents a challenge for environmental, human biomonitoring, metabolism, and toxicity studies.^25,35,36^ For example, unknown OH-PCB are frequently detected in environmental and biological samples.^36−43^ Computational approaches can facilitate the identification and quantification of OH-PCBs in environmental and biological samples. However, no method is currently available for identifying and quantifying these metabolites in any matrix.

Computational models trained with experimental observations represent an alternative approach for the nontarget analysis of diverse groups of chemicals. For example, models have been developed to predict the retention times and response factors of PCBs,^44,45^ polybrominated diphenyl ether,^46^ and human endogenous metabolites.^47^ In silico predictions can simulate the MS/MS spectra of chemicals to support the identification of unknown compounds.^48^ Previously, unknown OH-PCBs were quantified in abiotic samples using the average response factor for the OH-PCB homolog group.^43^ We have previously shown that mono–OH-PCBs without authentic analytical standards can be identified by homolog group and quantified in PCB-contaminated sediment using a seminontargeted approach. However, because our method could not identify the substitution patterns, and could not identify dihydroxyl PCBs, it was of limited use for interpreting the metabolic products of PCB exposure in laboratory animals.^35^

In this study, we used 124 analytical mono/di-MeO-PCB standards to develop multiple linear regression (MLR) or random forest regression (RFR) models that predict the retention times and MS/MS response data of MeO-PCBs on a GC-MS/MS system. The predicted GC-MS/MS data were used to identify and quantify OH-PCB metabolites in samples from animal studies with toxicologically relevant PCBs.

## Experimental Section

### Laboratory Methods

This study used machine learning-based approaches to identify and quantify the OH-PCBs detected in biological samples from PCB disposition and toxicity studies. The biological samples investigated include a feces sample from a PCB disposition study with mice acutely exposed to an individual PCB congener (PCB 95) and a liver sample from a PCB disposition study with mice subchronically exposed to a human-relevant PCB mixture. Briefly, adult mice were exposed to PCB 95 (1.0 mg/kg), a neurotoxic PCB,^49−52^ in stripped corn oil or corn oil alone. Feces from dissected distal colon and rectum were collected 24 h after PCB 95 exposure for analysis. The liver sample was collected as part of a larger study assessing the effects of developmental exposure to a PCB mixture on multiple developmental outcomes.^53−55^ The biological samples were extracted following a published procedure^41,56,57^ and analyzed by GC-MS/MS. For details regarding the animal studies, the extraction, and GC-MS/MS analysis, see the Supporting Information.

#### Experimental Determination of RRTs and MS/MS Profiles

Because of the high chromatographic resolution, OH-PCBs are typically extracted from biological or environmental matrices, derivatized to MeO-PCBs, and analyzed by GC-MS/MS.^4,14,15,58^ We measured the RRTs and MS/MS profiles [expressed as the relative intensities of five multiple reaction monitering (MRM) transitions] of two MeO-PCB standard solutions (solution 1 containing 72 MeO-PCBs and solution 2 containing 52 MeO-PCBs; see Supporting Information for additional information) using an Agilent 7890B gas chromatograph equipped with an SPB-Octyl capillary column (30 m length, 250 μm inner diameter, 0.25 μm film thickness; Supelco, Bellefonte, PA, USA), an Agilent 7000D Triple Quad and an Agilent 7693 sampler. For additional details, see the Supporting Information.

### Model Development

The 2-fold goal of the model is to predict the identity and calculate the concentration of mono- and dihydroxy PCBs in laboratory samples. We used MLR and RFR machine learning-based algorithms to develop models for identifying and quantifying OH-PCBs. These models used experimental RRT and RRF data (the components of MS/MS profiles) as dependent variables and molecular descriptors (MDs) as predictors. For the generation of chemoinformatics-based MDs with the *rcdk* package^59^ and substitution pattern-based MDs from the structure of the 124 MeO-PCBs (Table S1), see the Supporting Information. All data analyses were performed in R (version 3.6.3).

#### Preliminary Data Inspection

Since the MLR, but not the RFR models, assume normal data distribution and homogeneity of data variance,^60^ a preliminary data inspection was performed on all data sets used to predict the RRTs and RRFs of MeO-PCBs with the MLR model. Inspection of diagnostic plots [i.e., normal probability plots (*Q*–*Q* plots) and residual vs fitted value plots] for the RRT predictions suggested that the assumptions of data normality and variance homogeneity were supported by the majority of the 112 observations in the training data sets (Figure S1).

The training data sets used for predicting RRFs revealed nonlinear relationships. Therefore, the measured RRFs were log-transformed to obtain normally distributed data and account for nonlinear relationships. Potential outlier observations were removed by Cook’s distance (CD) with the following cutoff: CD < 10-fold of averaged CD (assuming outliers have CDs substantially larger than the averaged CD by over an order of magnitude). As a result, 109 and 88 observations remained in the training data sets used to develop models to predict RRTs and RRFs. Coeluting MeO-PCBs in the training data set were removed for the prediction of RRFs.

#### MLR Model Development

We used a repeated 10-fold cross-validation strategy^61,62^ to train and internally validate the MLR models used to predict the RRTs or RRFs of MeO-PCBs. First, MLR modeling underwent a predictor selection step to minimize the number of predictors and enhance model stability without sacrificing model performance. This step was performed with the *stepAIC* function in the *MASS* package (https://cran.r-project.org/web/packages/MASS/index.html). Next, predictors were optimized stepwise with the Akaike Information Criteria (AIC) for variable selection. Based on this optimization step, ten out of 105 MDs were selected to predict RRTs (Table S2), and 16 to sixty-six out of 105 MDs were used to predict the RRFs of the five MS transitions.

The observations from each data set were randomly divided into ten groups. Nine groups were used as the training data set, and the remaining data set was used for internal testing. The model training and testing were performed ten times to ensure that each group was used once as the testing data set. The data grouping, model training, and internal testing were repeated five times to avoid biases in the initial random grouping of the data sets. Finally, MLR models with predictor coefficients and their deviations at the least root-mean-square error (RMSE) were generated to predict RRTs or RRFs. The MLR models were evaluated by *R*^2^ (RSQ), mean absolute error (MAE), and RMSE between the predicted and measured value and the prediction interval at the 95% confidence level.

#### RFR Model Development

Initially, RFR models were constructed to predict RRTs or RRFs with all MDs as independent variables and experimental RRTs or RRFs as dependent variables using the R package *randomForest*. Approximately two-thirds of the MeO-PCBs were randomly selected as the internal training data set, and the rest were used as the internal testing data set. An importance value was assigned to each MD to evaluate its contribution to the prediction model. The model construction was repeated 100 times with randomly selected data sets to identify the top six ranked MDs for each iteration. The MDs that appeared >50 times in these RFR models were chosen for further predictions (Table S3).

Subsequently, the parameters in the random forest algorithms, *ntree* (i.e., number of trees to grow) and *mtry* (i.e., number of variables randomly sampled as candidates at each split), were optimized from 100 to 1000 with a step size of 100 for *ntree* and from one variable to the total number of variables for *mtry*. The two parameters were permutated to form a set of parameter combinations. The performance of each parameter combination was evaluated using the RMSE. The parameter combination with the smallest RMSE was used to construct the final prediction model. For information on the optimized *ntree* and *mtry* for predicting RRFs, see Table S3. In the final model prediction step, the optimized MDs (predictors) and RF parameters were used to predict the RRTs or RRFs of the MeO-PCBs with the RFR models.

#### Model Validation

The MLR and RFR models were validated with external data sets containing 12 MeO-PCBs for RRTs predictions and 11 MeO-PCBs for RRFs predictions (data for one MeO-PCBs was removed because it was below the detection limit) (see Table S1).

### Candidate Ranking in Identifying Unknown OH-PCBs (as Methylated Derivatives)

Preliminary data analysis suggested that MeO-PCB isomers (i.e., varied chlorine or methoxy substitution patterns) have drastically different responses for the same MRM transition in the GC-MS/MS analysis (Figure S2). Therefore, in addition to the predicted RRT, we used the predicted MS/MS data, consisting of the relative intensities of five fragment ions, to rank MeO-PCBs isomers derived from the same PCB congener or homolog to identify OH-PCBs in animals samples (i.e., feces and liver). For more details regarding the candidate ranking strategy, see the Supporting Information.

## Results and Discussion

### Prediction of RRTs of MeO-PCBs

The identification of OH-PCBs in environmental and biological samples is challenging because of the large number of possible OH-PCBs and the structural similarity of OH-PCB metabolites of a specific PCB congener (e.g., PCB 95 or PCB 28). Therefore, it is unlikely that a single approach can achieve unambiguous identification of specific OH-PCB isomers; however, machine learning methods have the potential to aid in the identification of OH-PCB isomers.

**Figure 1:**
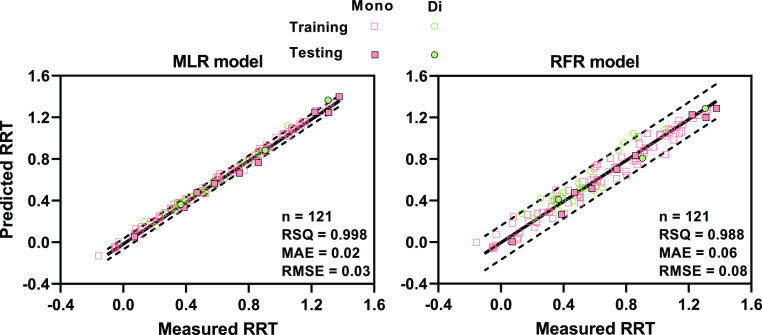
A multiple linear regression (MLR) model provided a better estimation of the RRTs of MeO-PCBs compared to the random forest regression (RFR) model. The model training data sets were constructed with the measured RRTs and molecular descriptors of 87 mono-MeO-PCBs and 22 di-MeO-PCBs. The testing data set contains the measured RRTs and molecular descriptors of nine mono-MeO-PCBs (mono- to nona-chlorinated) and three di-MeO-PCBs (di-, tetra-, or octa-chlorinated). The dash lines indicate the borders of the prediction interval with a 95% confidence level.

We developed MLR and RFR models to predict the RRTs of MeO-PCBs on a GC-MS/MS system equipped with an SPB-Octyl column. Both models provide good approximations of the RRTs of MeO-PCBs, with R^2^ values (derived from linear regressions between the measured and predicted values) greater than 0.98 (Figure 1) and with randomly distributed residuals (Figure S3). The MLR model with 10 predictors performed better, with a narrower prediction interval and lower RMSE, than the RFR models with the same number of predictors. The absolute difference between measured and predicted retention times was within 1 min for 87% observations (*n* = 121) in the MLR model predictions. This finding is not surprising because statistically significant linear relationships can be readily established between the predictors and the RRTs of MeO-PCBs in the MLR development, with *p* < 0.05 for all 10 predictors (Table S2).

The MLR models developed with data from the SPB-octyl column slightly underestimate the RRTs of MeO-PCBs collected with a different GC column (DB-1701) by overall 2% (Figure S4, data was collected in a previous study), indicating a likely column flexibility, at least for poly(n-octyl/methyl siloxane) phase columns. In addition to predicting the RRTs of MeO-PCBs, the MLR models can also provide reasonable estimates of the RRTs of PCBs collected under identical conditions but with a physically different instrument (Figure S5). This finding indicates that slight changes in chemical structure (e.g., with or without the methoxy group) and a physically different instrument are unlikely to affect the model applications. However, the same commercially available internal standards and similar instrument conditions are recommended to apply the models to other problems. MLR models performed better than analogous RFR models for the prediction of RRTs of MeO-PCBs on a DB-1701 column and RRTs of PCBs on an SPB-Octyl column (Figure S5).

This study is the first report of predictive models for OH-PCBs, but both MLR and RFR models are widely used for predicting the retention times of chemicals on GC or LC systems. For example, an MLR model with five PCB molecular descriptors (selected from topological descriptors, geometric descriptors, electronic descriptors, and calculated physical property descriptors) predicted the RRT of PCBs on a GC column with a relative standard deviation of 1.7%.^45^ Analogously, a five-variable MLR model with molecular electronegativity distance vectors of PCBs predicted the RRT of the PCBs with an RMSE of 0.0152 (or an MAE of approximately 1.90 min in retention time).^44^ Retention times of chemicals were also predicted with RFR models on LC columns to facilitate the identification of unidentified peaks in untargeted metabolomics, with MAEs of 0.78 min (20% in mean relative error) and 0.57 min (13% in mean relative error) for hydrophilic interaction chromatography and reverse-phase LC columns, respectively.^47^ The retention times of polybrominated diphenyl ethers and their methoxylated metabolites on a GC column were predicted with a lower accuracy by linear regression with the melting points.^46^ Our MLR model with 10 predictors obtained comparable accuracy as above in predicting retention times of MeO-PCBs with an overall MAE of 0.55 min (n = 121) (Figure S3). However, the accuracy of the RRT predictions with this and other models does not meet the RRT variation tolerance recommended by the European Commission for identifying chromatographic peaks (i.e., 0.5% and 2.5% for GC and LC peaks, respectively).^63^ Therefore, other identifiers, such as MS/MS profiles, are needed to identify unknown peaks.

### Prediction of MS/MS Profiles of MeO-PCBs

**Figure 2:**
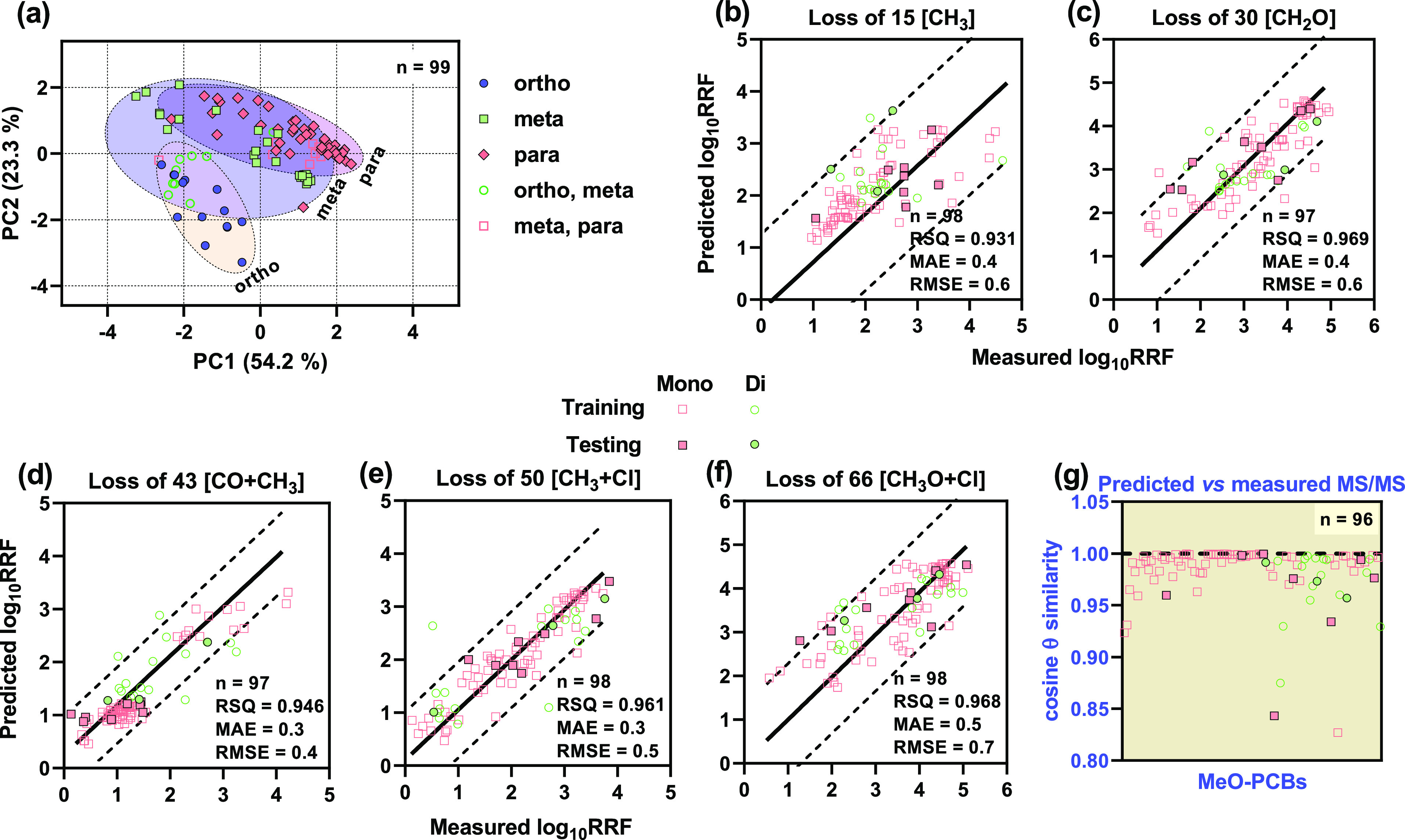
Responses of five fragmentations (i.e., the loss of 15 [CH_3_], 30 [CH_2_O], 43 [CH_3_ + CO], 50 [CH_3_ + Cl], and 66 [CH_3_O + Cl]) of the MeO-PCBs varied with the position (*ortho*, *meta*, or *para*) of the methoxy group, as revealed by (a) a principal component analysis (PCA). (b–f) Random forest regression model with molecular descriptors as predictors provided reasonable estimations of the responses of five fragmentations studied. The model training and testing data sets were constructed with the MS/MS data (expressed as the relative response factors) from 88 and 11 observations, respectively. The dash lines indicate the borders of the prediction interval with a 95% confidence level. (g) The similarity coefficient cos θ showed agreement between predicted and measured MS/MS profiles of MeO-PCBs.

Principal component analysis and a violin plot of the MS/MS profiles of 99 mono- or di-MeO-PCBs suggested that their MS/MS data vary significantly with the position (i.e., ortho, meta, or para) of the methoxy group on the biphenyl moiety (Figures 2a and S2). Notably, higher signals were observed for the loss of 50 (i.e., [CH_3_+Cl]) for MeO-PCBs with ortho methoxy groups. On the other hand, meta- or para-methoxylated PCBs are more likely to fragment with the loss of 43 [CH_3_ + CO]. Since the loss of [CO] requires the opening of the MeO-substituted benzene ring, it is likely that the meta- and para-methoxylated PCBs chemically have a more favorable configuration for ring opening than that of ortho-methoxylated PCBs, as illustrated in Figure S6. This substitution pattern-dependent response suggests that MS/MS data can be used to assign the structure (i.e., ortho vs meta or para-methoxy) of an unknown peak. Likewise, MS/MS responses were previously used to identify MeO-PCB 28 isomers formed in rats exposed to PCB 28.^64^

We predicted the MS/MS data of MeO-PCBs (expressed as the relative levels of the signals of the five fragmentations investigated) using RFR models coupled with MDs as predictors. The prediction of the RFR model, but not the MLR model, provided good approximations of the response for all five fragmentations, with MAE ranging from 0.3 to 0.5 log units (Figure 2b–f). However, better estimations with a narrower prediction interval and lower MAE were obtained when predicting the RRFs associated with the loss of 43 or 50, likely because MeO-PCBs have higher responses generated through these two fragmentations. Importantly, the predicted MS/MS profiles were similar to the experimental data, with the similarity coefficient^65^ cos θ > 0.95 for 92% of the 96 MeO-PCBs investigated (Figure 2g) (cos θ = 1 indicates that the MS/MS profiles are an exact match, cos θ = 0 indicates different profiles).

Since MS/MS data carry fragment information that can be used to identify unknown peaks, several programs (e.g., MetFrag,^66^ CFM-ID,^48^ and CSI:FingerID^67^) have been developed to predict the MS/MS data from the corresponding molecular structure. These programs were primarily designed for soft ionization systems, such as electrospray ionization (ESI), and provide no meaningful intensity values for the fragmentation of MeO-PCBs on a GC-MS/MS system with electron ionization (EI). Thus, the information provided by these software packages does not facilitate the identification of MeO-PCB isomers. CFM-ID has the option to simulate EI-MS spectra, but not EI-MS/MS spectra. Consequently, the intensity information predicted by this approach in either EI-MS or ESI-MS/MS mode poorly reflects the experimental EI-MS/MS intensities in part because the CFM-ID program was originally not trained with reference MS spectra of MeO-PCBs (Figure S7). Our machine-learning models were trained and externally validated with experimental MS/MS data of 124 mono/di-MeO-PCBs and, for the first time, allow the quantitative prediction of the MS/MS data of MeO-PCBs for which no authentic analytical standards are available. The predicted MS/MS data provide an additional dimension assisting in the identification of unknown MeO-PCB peaks.

### Quantification of MeO-PCBs with the Predicted RRFs

**Figure 3:**
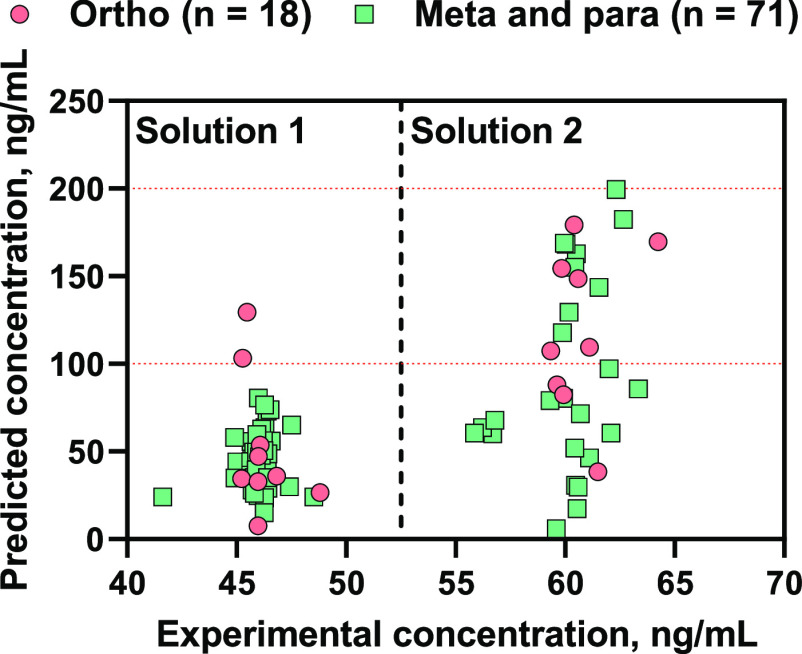
A comparison of the levels of MeO-PCBs quantified by predicted relative response factors (RRFs) with experimental values. The RRFs of MeO-PCBs were predicted with the random forest regression model coupled with the molecular structures. The ortho-methoxylated PCBs were quantified with RRFs predicted for the loss of 50 [CH_3_ + Cl], and the meta- and para-methoxylated PCBs were quantified with RRFs predicted for the loss of 43 [CH_3_ + CO]. Two MeO-PCBs standard mixtures (solutions 1 and 2) with concentrations of 47 and 60 ng/mL, respectively, were used.

After the structural identification of an unknown MeO-PCB with the predicted retention time and MS/MS data, the unknown peak can be quantified with predicted RRFs. Since the MS/MS responses of MeO-PCBs depend on the position of the methoxy group on the biphenyl moiety (Figure S2), we used signals of the respective transitions for the loss of 50 [CH_3_ + Cl] to quantify ortho-methoxylated PCBs and the loss of 43 [CH_3_ + CO] to quantify meta- or para-methoxylated PCBs. The levels of 89 MeO-PCBs (di-MeO-PCBs with both ortho- and meta/para-methoxy groups were excluded) predicted with this approach were within a 2-fold difference for 85% observations and within a 3-fold difference for all observations (Figure 3). These results demonstrate that the predicted RRFs allow a good approximation of the levels of OH-PCBs (as methylated derivatives) within 1 order of magnitude.

The RRFs of mono-MeO-PCBs for GC-MS analyses in the selected ion monitoring (SIM) mode have been predicted with a quadratic model using the number of chlorine atoms as a predictor.^35^ This model was trained with one of the standard mixtures (solution 1) used in this study (Figure 3). The RRFs predicted by the quadratic model were verified by quantifying 12 mono-MeO-PCBs with values ranging from 0.8 to 2 times of the actual concentrations. The RRFs predicted by our RFR model estimated the levels of 96% of the solution 1 authentic analytical standards (*n* = 54, coeluting and di-MeO-PCBs were not included) within a 2-fold difference (0.5–2 times of the actual concentrations) and, thus, have similar accuracy as the earlier model. This observation is not surprising because the use of MRM signals increases the complexity of the modeling while increasing the selectivity in identifying unknowns. A lower accuracy was observed when estimating the levels of the second standard solution (solution 2), likely because this standard solution contained most of the di-MeO-PCBs included in this study.

### Characterization of OH-PCBs Using Predicted RRTs, MS/MS Data, and RRFs

**Figure 4:**
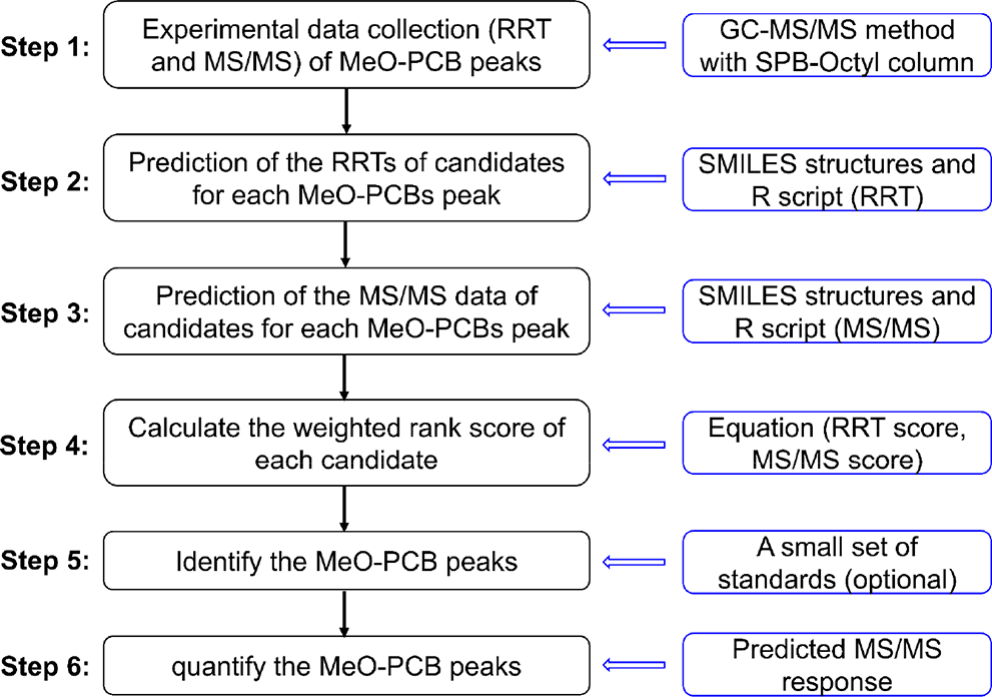
Proposed workflow for the identification and quantification of OH-PCBs (analyzed as methylated derivatives) using predicted retention times (RRT) and MS/MS responses.

The flowchart in Figure 4 illustrates how we propose to use the predicted RRT and MS/MS data to aid in the identification and quantification of OH-PCB metabolites (as methylated derivatives) in environmental or biological samples. Step 1: Sample extracts containing OH-PCBs are derivatized and analyzed by GC-MS/MS, as described in the Experimental Section, to collect experimental RRT and MS/MS data of the OH-PCBs. Step 2: For each OH-PCB metabolite peak, the RRTs of all possible MeO-PCB derivatives, as their SMILES structures, are predicted with our RRT prediction model. Step 3: The MS/MS data of all possible structures of an OH-PCB metabolite peak, also as their SMILES structures, are predicted with our MS/MS prediction model. Step 4: The weighted rank scores of all candidate structures are calculated (see the Supporting Information). Step 5: Identify the OH-PCB metabolite peaks based on the weighted rank scores. If available, a small set of MeO-PCB standards can be used to assist with the identification of the OH-PCB isomers. Step 6: The OH-PCB peaks are integrated and quantified using the predicted MS/MS responses. A data set containing the detailed user manual of these steps, example data and the R codes are publicly available in Iowa Research Online at http://doi.org/10.25820/data.006179. The following section demonstrates the application of this approach to facilitate the identification and quantification of OH-PCB 95 in mouse feces and OH-PCB 28 in mouse liver. Since the model predictions were originally trained using experimental data obtained with standard solutions, these predictions facilitate the availability of standard retention times and MS/MS response factors independent of the sample matrix. OH-PCBs in any sample matrix can be theoretically identified and quantified with the predicted standard retention times and MS/MS data as long as necessary sample preparation procedures were performed, as described in this and other studies.^4,14,15,58^

#### Analysis of OH-PCB 95 in the Feces of a Mouse Exposed to PCB 95

**Figure 5:**
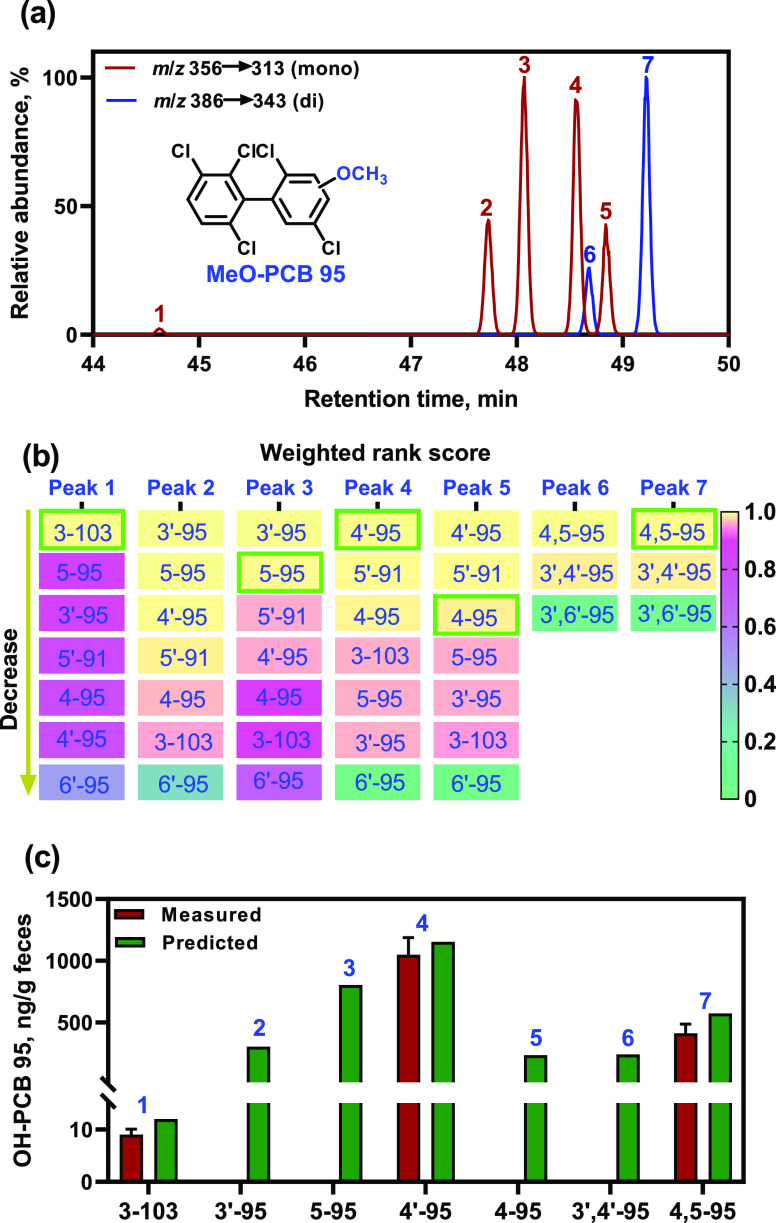
(a) GC-MS/MS chromatograms indicate the presence of five peaks (peaks 1, 2, 3, 4, and 5) of monohydroxylated metabolites and two peaks of dihydroxylated metabolites (peaks 6 and 7) in a feces sample from a mouse orally exposed to PCB 95. The OH-PCBs were analyzed as methylated derivatives. (b) Possible candidates for each peak were proposed and ranked based on their weighted scores calculated with measured and predicted retention times and MS/MS data. The candidate structures of OH-PCB in this and the following figures are abbreviated with the position of the OH group plus their PCB number, for example 4–95. The candidates in green borders was unambiguously identified with an authentic standard. (c) The agreement between measured and predicted levels of the OH-PCB 95 metabolites (i.e., 3–103, 4′-95, and 4,5-95) supports the quantification of OH-PCBs with a predicted relative response factor. The abbreviations and the corresponding structures of the MeO-PCB 95 metabolites are provided in Figure S8.

PCB 95 and its metabolites are potentially neurotoxic.^49−52^ Because metabolites of higher chlorinated PCBs are excreted with the feces,^68^ we investigated OH-PCBs in a feces sample from a mouse exposed to PCB 95. We detected 5 peaks (peaks 1, 2, 3, 4, and 5) with the MS transition *m*/*z* 356 → 313, corresponding to pentachlorinated mono-MeO-PCBs, and 2 peaks (peaks 6 and 7) with the MS transition *m*/*z* 386 → 343, corresponding to pentachlorinated di-MeO-PCBs, in the extract of feces from a mouse exposed to PCB 95 (Figure 5a). The possible mono-MeO-PCB 95 and selected di-MeO-PCB 95 that are likely formed in PCB metabolism studies, for example, metabolites with two methoxy groups ortho or para to each other, are shown in Figure S8. The MeO-PCB 95 candidates were ranked based on their weighted rank scores calculated from the predicted and experimental RRT and MS/MS data (Figure 5b).

Overall, the model correctly suggested the position of methoxy groups (ortho, meta, or para). Briefly, peaks 1, 4, and 7 were correctly identified based on the weighted ranking scores as 3–103 (1,2-shift product), 4′–95, and 4,5–95, respectively. The weighted ranking scores suggested that peaks 3 and 5 correspond to a meta- and para-hydroxylated metabolite (3′–95 and 4′–95, respectively). Based on the elution order of authentic analytical standards of MeO-PCB 95 analyzed on the same GC column (SPB-Octyl) (Figure S9), peaks 3 and 5 correspond to meta- and para-hydroxylate metabolites (5–95 and 4–95, respectively). These two correct identifications ranked within the top 3 candidates (Figure 5b). Peak 2 was predicted to be 3′–95. This structural assignment requires confirmation with an analytical standard.

Peak 7 was correctly identified by the weighted rank scores as 4,5-PCB 95. The model also identified peak 6 as 4,5–95, another catechol metabolite; however, peak 6 likely corresponds to a different catechol metabolite, 3′,4′–95, as suggested by the top 2 candidate. This identification is consistent with the preferential formation of PCB catechol metabolites in PCB metabolism studies.^23−25^ Finally, PCB 95 metabolites were quantified with their predicted RRFs. The predicted and experimental levels of the metabolites with available authentic standards (i.e., peaks 1, 4, and 7) showed good agreement across concentration levels over 3 orders of magnitude (9–1048 ng/g) (Figure 5c). Thus, the predicted RRF allows a reasonable approximation of the levels of PCB 95 metabolites for which no authentic analytical standards are available. The MS/MS responses of authentic standards of 5–95 (peak 3) and 4–95 (peak 5) were measured with a different GC-MS/MS method and were not included in the comparisons with the predicted levels in Figure 5c.

The identification of PCB 95 metabolites using our model in combination with authentic analytical standards increases the confidence in the identification of unknown OH-PCB 95 metabolites in the feces sample from this study, but also earlier studies investigating the metabolism of PCB 95. For example, an unknown MeO-PCB 95 peak was detected in metabolism studies with rat cytochrome P450 enzymes,^39^ rat and human liver microsomes^36,41^ and in vivo disposition studies in rodent models.^37,38,40^ In these previous studies we tentatively identified this unknown peak, which eluted before 5–95 on an SPB-1 column, as 3′-95. Our present study confirms this tentative identification of 3′-95 despite the difference in GC column stationary phases. Similarly, earlier metabolism studies with human liver microsomes or rats *in vivo* reported an unknown dihydroxylated PCB 95 metabolite peak (as its methylated derivative) that eluted before 4,5–95 on the SPB-1 column.^36,37^ In the absence of an authentic standard, the model predictions provide an additional line of evidence supporting the identification of this metabolite as 3′,4′–95, another PCB 95 catechol metabolite.

#### Analysis of OH-PCB 28 in the Liver of a Mouse Exposed to a Neurotoxic PCB Mixture

**Figure 6:**
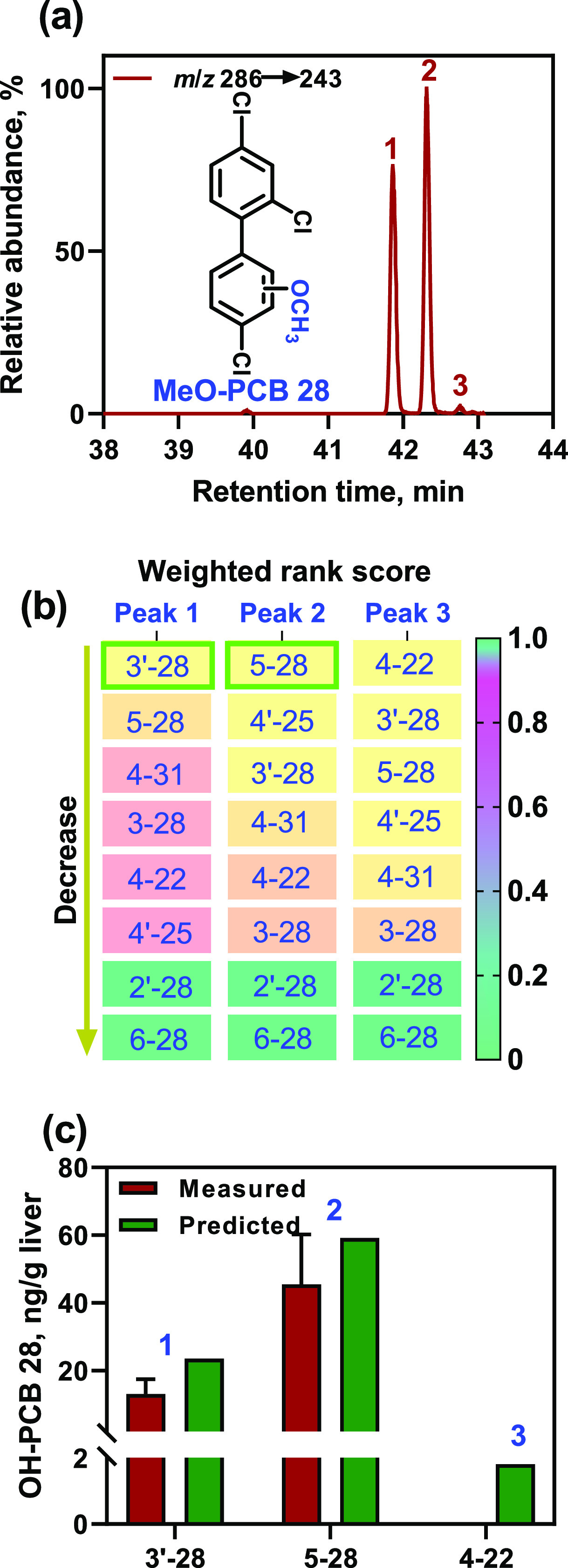
(a) GC-MS/MS chromatograms support the formation of three peaks (peaks 1, 2, and 3) of monohydroxylated metabolites of PCB 28 in a liver sample collected from a mouse exposed throughout gestation and lactation to a PCB mixture (6 mg/kg/day) containing PCB 28 as a major component. (b) Possible candidate for each metabolite peak were propose and ranked with their weighted scores calculated with measured and predicted retention times and MS/MS data. The candidates in green borders were unambiguously identified with an authentic standard. (c) The agreement between measured and predicted levels of the OH-PCB 28 metabolites supports the quantification of OH-PCBs with a predicted relative response factor. The abbreviations and the corresponding structures of the MeO-PCB 28 metabolites are provided in Figure S10.

We also investigated metabolites of PCB 28 in the liver from a mouse exposed during gestation and lactation to a PCB mixture.^53−55^ Based on the MS transition *m*/*z* 286 → 243, we identified three trichlorinated MeO-PCB peaks (peaks 1, 2, and 3) corresponding to monohydroxylated metabolites of PCB 28 (Figure 6a). Based on the experimental and predicted RRT and MS/MS data, the weighted rank scores of all possible MeO-PCB 28 candidates (Figure S10) were calculated for the three MeO-PCB 28 peaks (Figure 6b). The top candidates for peaks 1, 2, and 3 were 3′–28, 5–28, and 4–22 (a 1,2-shift product of PCB 28), respectively. The identification of peaks 1 and 2 was subsequently confirmed with authentic standards. Using a small set of MeO-PCB 28 standards, we confirmed that Peak 3 does not correspond to 2′–28, 3–28, or 4′–25 (another 1,2-shift product of PCB28). Likely, Peak 3 was correctly identified as 4–22 by our model; however, confirmation with an authentic standard is still needed if this minor metabolite becomes a concern. The three peaks of PCB 28 metabolites were quantified with their predicted RRFs. As with the PCB 95 metabolites above, the OH-PCB levels calculated with the predicted RRFs are in good agreement with the experimental levels of the two metabolites for which authentic analytical standards are available (i.e., 3′–28 and 5–28) (Figure 6c).

Our predictions also enable a tentative identification of unknown metabolites observed in an earlier study. Briefly, two major, meta-hydroxylated PCB 28 metabolites and two minor para-hydroxylated PCB 28 metabolites (analyzed as methylated derivatives) were eliminated with the feces of rats exposed intraperitoneally to PCB 28.^64^ One meta-hydroxylated PCB 28 metabolite was identified as 5–28 with a synthetic standard on a GC-MS equipped with a BP-5 column. The other unidentified, meta-hydroxylated metabolite eluted at an earlier retention time. Based on the elution order, we hypothesize that this metabolite corresponds to 3′–28 (peak 1) observed in this study (Figure 6a), irrespective of the different GC columns used. The two para-hydroxylated PCB 28 metabolites were 1,2 shift products and remain unidentified because of the lack of analytical standards. Similar to this study, one of the unknown para-hydroxylated PCB 28 metabolites likely is 4–22.

The PCB metabolism studies described above highlight the complexity of the metabolism of PCBs and the challenges associated with the identification of the PCB metabolites, which depend on the availability of authentic analytical standards. The proposed strategy using machine learning-based model predictions can significantly advance identifying and quantifying unknown OH-PCBs, especially in combination with a small set of authentic analytical standards. Notably, the predicted top candidate can suggest if the methoxy group is in the ortho, meta, or para position. Even if the top candidate is not the true compound, knowing the position of the methoxy substituent enables a targeted synthesis of authentic analytical standards. Additional studies are needed to demonstrate that our machine learning approach can facilitate the identification of OH-PCB metabolites in environmental and biological samples.

## Supporting Information Available

Sources and structures of MeO-PCBs, GC-MS/MS parameters, the candidate ranking algorithm, details regarding sample collection and extraction from animal experiments, optimal predictors and their linear coefficients and *p*-values to RRTs and MS/MS data of MeO-PCBs, diagnostic plots for model development, predominant pathways and data of the MeO-PCB fragmentations, model predictions of RRTs collected with a different GC column and RRTs of PCBs, CFM-ID predictions, and the structures, and abbreviations and SMILES structures of MeO-PCB 95 and MeO-PCB 28 (PDF)
