# Supplementary material for: Machine Learning-Assisted Identification and Quantification of Hydroxylated Metabolites of Polychlorinated Biphenyls in Animal Samples
Source: Environ Sci Technol. 2022 Sep 1;56(18):13169–78. doi: 10.1021/acs.est.2c02027 (PMC9573770; doi:10.1021/acs.est.2c02027)
Supplement: Supplementary file 1 — es2c02027_si_001.pdf [file es2c02027_si_001.pdf]

SUPPORTING INFORMATION

MACHINE LEARNING-ASSISTED IDENTIFICATION AND QUANTIFICATION OF HYDROXYLATED METABOLITES OF POLYCHLORINATED BIPHENYLS IN ANIMAL SAMPLES

Chun-Yun Zhang[1], Xueshu Li[1], Kimberly P. Keil Stietz[2], Sunjay Sethi[2], Weizhu Yang[3], Rachel F. Marek,[4] Xinxin Ding[3], Pamela J. Lein[2], Keri C. Hornbuckle[4], Hans-Joachim Lehmler[1,*]

[1]Department of Occupational and Environmental Health, The University of Iowa, Iowa City, Iowa 52242, United States. [2]Department of Molecular Biosciences, School of Veterinary Medicine, University of California Davis, Davis, California 95616, United States. [3]Department of Pharmacology and Toxicology, College of Pharmacy, University of Arizona, Tucson, Arizona

85721, United States. [4]Department of Civil and Environmental Engineering and IIHR Hydroscience and Engineering, The University of Iowa, Iowa City, IA 52242, United States.

[*]Corresponding Author:
Dr. Hans-Joachim Lehmler
The University of Iowa
Department of Occupational and Environmental Health
University of Iowa Research Park, #164 MTF
Iowa City, IA 52242-5000
Phone: (319) 335-4981
Fax: (319) 335-4290
e-mail: hans-joachim-lehmler@uiowa.edu

Number of pages: 32
Number of tables: 3
Number of figures: 10

Table of Contents

Chemicals	S4
Gas chromatography-tandem mass spectrometric (GC-MS/MS) determination of relative retention times (RRTs) and MS/MS data of methoxylated PCBs (MeO-PCBs)	S4
Molecular descriptors	S5
Candidate ranking algorithm with the predicted and measured RRTs and MS/MS data of MeO-PCBs	S6
Animal experiments	S8
Extraction of the hydroxylated PCBs from samples collected in animal studies	S10
**Table S1.** List of methoxylated PCBs (MeO-PCBs) used for model training and external testing and their abbreviations and SMILES structures	S12
**Table S2**. List of the optimal predictors and their linear coefficients and p-values that were obtained in the multiple linear regression (MLR) model development to predict the relative retention time (RRT) of MeO-PCBs	S16
**Table S3.** List of the optimal predictors and parameters obtained in the random forest regression (RFR) model development to predict MS/MS data (expressed as relative levels of five MS transitions) of MeO-PCBs	S17
**Fig. S1.** Diagnostic plots [(a) residuals versus fitted values, (b) scale location, (c) normal Q–Q and (d) residuals versus leverage] for multiple linear regression for predicting relative retention times (RRT) of MeO-PCBs reveal no non-linear relationship between the predictors and the RRTs and support normal data distribution and homogeneity of data variance in the model development	S18
**Fig. S2.** Five representative MS/MS responses associated with mass losses of 15 [CH_3], 30 [CH_2O], 43 [CH_3+CO], 50 [CH_3+Cl] and 66 [CH_3O+Cl] of MeO-PCBs varied with the substitution positions (ortho-, meta- and para-) of the methoxy group.	S19
**Fig. S3.** Plots of absolute differences between measured and predicted RRTs versus experimental retention times revealed randomly distributed residuals in MLR and RFR model predictions.	S20
**Fig. S4.** Both (a) multiple linear regression (MLR) and (b) random forest regression (RFR) models developed with SPB-Octyl column slightly underestimated the relative retention times (RRTs) of MeO-PCBs measured with a DB-1701 column	S21
**Fig. S5.** The (a) multiple linear regression (MLR) model developed for predicting the relative retention times (RRTs) of MeO-PCBs can also provide reasonable estimations of the RRTs of PCBs collected with the same a SPB-Octyl GC column with a physically different instrument (SPB-Octyl) and performed better than (b) the random forest regression (RFR) model in predicting the RRTs of PCBs	S22
**Fig. S6.** The predominant fragmentation pathways of MeO-PCBs with different substitution positions (ortho, meta and para) of the methoxy group.	S23

Fig. S7. The comparison between measured and predicted spectra suggests CFM-ID with either EI-MS or ESI-MS/MS modules poorly simulated the fragmentation of MeO-PCBs. S24

Fig. S8. The abbreviations and their corresponding structures of all possible MeO-PCB 95 metabolites S25

Fig. S9. GC-MS/MS chromatograms of the authentic standards of mono- and di-hydroxylated metabolites of PCB 95 (analyzed as methylated derivatives). S26

Fig. S10. The abbreviations and their corresponding structures of all possible mono-MeO-PCB 28 metabolites S27

References S28

Chemicals. Seventy-two methoxylated PCBs (MeO-PCBs) (70 mono-MeO-PCBs and two di-MeO-PCBs as standard Solution 1) were purchased from AccuStandard (New Haven, CT, USA) and Wellington Laboratories (Guelph, ON, Canada). Fifty-two MeO-PCBs (28 mono-MeO-PCBs and 24 di-MeO-PCBs as standard Solution 2) were synthesized with Suzuki coupling reaction between a suitable benzene boronic acid and a methoxylated bromochlorobenzene,[1, 2] and authenticated as described.[3-9] The suite of mono-methoxylated compounds included 12 mono-, 11 di-, 14 tri-, 14 tetra-, 19 penta-, 11 hexa-, 10 hepta-, 6 octa-, and 1 nona-chlorinated MeO-PCB congeners. The di-methoxylated standards included 3 mono-, 15 di-, 1 tri-, 1 tetra-, 2 penta-, 3 hexa-, and 1 octa-chlorinated di-MeO-PCB congeners. Two standard solutions (Solution 1 and Solution 2) containing 72 and 52 MeO-PCBs (about 50 ng/mL of each MeO-PCB) were prepared for this study. These MeO-PCB derivatives correspond to hydroxylated PCB (OH-PCB) metabolites and are abbreviated based on the nomenclature for PCB metabolites suggested by Maervoet and co-workers.[10] For a full list of the MeO-PCBs used in this study, including their abbreviations and SMILES structures, see Table S1. 2,4,6-Trichloro-(2',3',4',5',6'-$^{2}H_{5}$)-biphenyl (d_5-PCB 30) and 2,2',3,4,4',5,6,6'-octachlorobiphenyl (PCB 204) were used as internal standards and purchased from Cambridge Isotope Laboratories (Andover, MA, USA) and AccuStandard (New Haven, CT, USA), respectively. Standards for the analysis of OH-PCBs in biological samples, including 4′-OH-2,3,3′,4,5,5′-hexachlorobiphenyl (4′-159) and PCB 117, were also obtained from AccuStandard.

Gas chromatography-tandem mass spectrometric (GC-MS/MS) determination of relative retention times (RRTs) and MS/MS data of methoxylated PCBs (MeO-PCBs). An Agilent 7890B gas chromatograph equipped with an SPB-Octyl capillary column (30 m length, 250 µm inner diameter, 0.25 µm film thickness; Supelco, Bellefonte, PA, USA) and an Agilent

7000D Triple Quad MS system was used to analyze the MeO-PCBs for the obtaining of their RRTs and MS/MS data (expressed as the relative intensities of five MRM transitions). The electron ionization (EI) source operated at an energy of 70 eV. Helium was used as the carrier gas at a constant flow rate of 0.8 mL/min. The temperature program started at 45 ^{o}C, hold for 2 min, increased to 75 ^{o}C by 100 ^{o}C/min, hold for 5 min, increased to 150 ^{o}C by 15 ^{o}C/min, hold for 1 min, increased to 280 ^{o}C by 2.5 ^{o}C/min, and hold for 5 min. The transfer line temperature was 280 ^{o}C. The temperatures of the MS source and quadrupoles were 250 ^{o}C and 150 ^{o}C, respectively. Data for all MeO-PCBs were collected in the MRM mode by recording the following MS transitions at a collision energy of 25 eV: The loss of 15 [CH_3], the loss of 30 [CH_2O], the loss of 43 [$CO+CH_3$], the loss of 50 [CH_3+Cl] and the loss of 66 [CH_3O+Cl]. These MS transitions were selected based on published fragmentation patterns of MeO-PCBs.[11-13]

The RRTs of MeO-PCBs were calculated based on the retention times of the analyte (R_t) and the two internal standards (R_t^{dPCB30} for d_5-PCB 30 and R_t^{PCB204} for PCB 204) with the equation: $RRT = \left(R_t^{MeOPCB} - R_t^{dPCB30}\right)/\left(R_t^{PCB204} - R_t^{dPCB30}\right)$. The RRTs of d_5-PCB 30 and PCB 204 are 0 and 1, respectively, regardless of the retention time shift across sample batches. The RRFs of MeO-PCBs were calculated as $RRF = \frac{A_{dPCB30}/C_{dPCB30}}{A_{MeOPCB}/C_{MeOPCB}}$, where A_{dPCB30} and A_{MeOPCB} are the peak areas of the internal standard (d_5-PCB 30) and MeO-PCBs, respectively. C_{dPCB30} and C_{MeOPCB} are the concentrations of the internal standard (d_5-PCB 30) and MeO-PCBs, respectively.

Molecular descriptors. Molecular descriptors (MDs) of the 124 mono/di-MeO-PCBs, including chemoinformatics and substitution pattern-based MDs, were generated as described below. For more information regarding the 124 mono/di-MeO-PCBs, see Table S1.

Chemoinformatics-based MDs. We used the R package *rcdk*,[14] an interface to access the Chemical Development Kit (CDK) libraries, to calculate the chemoinformatics-based MDs, for example, Fsp3, XLogP and WPATH, of all 124 mono/di-MeO-PCBs used in this study. Briefly, structures of all MeO-PCBs were drawn with ChemBioDraw Ultra 14.0 (PerkinElmer, Waltham, MA, USA) and exported as SMILES structures. The SMILES structures of the MeO-PCBs were subsequently used to extract the MDs with the *rcdk* package. A total of 287 MDs were calculated for each MeO-PCB congener across all molecular descriptor categories. MDs with values missing for more than 50 % of the compounds were not included in further analyses. All other missing MD values were imputed using the KNN algorithm with the R package *impute*. The MDs with the same value for all compounds were also removed from the dataset. This approach resulted in a final list of 99 chemoinformatics-based MDs for each MeO-PCB congener.

Substitution pattern-based MDs. We also generated MDs that describe the MeO and Cl substitution patterns of the MeO-PCBs. These MDs describe the position of the substituents on the benzene rings, including the numbers of para- ($N_{MeO,para}$), meta- ($N_{MeO,meta}$), and ortho-substituted methoxy groups ($N_{MeO,ortho}$), and the numbers of para- ($N_{Cl,para}$), meta- ($N_{Cl,meta}$), and ortho-substituted chlorine atoms ($N_{Cl,ortho}$).

Candidate ranking algorithm with the predicted and measured RRTs and MS/MS data of MeO-PCBs. After determining the possible MeO-PCB structures (candidates) based on the molecular weight of the molecular ion and the featured product ions in the MRM method, the following approach was used to rank candidates of an unknown MeO-PCB peak:

Initially, RRT scores (S_T) were calculated for all possible MeO-PCB candidates of a specific molecular weight. We used the reciprocal of the absolute difference between the measured

($RRT_{measured}$) and predicted RRT ($RRT_{predicted}$) to score potential candidates (i.e., the smaller the difference, the higher the score). The initial score was then normalized with the maximal ($S_{T,max}$) and the median ($S_{T,med}$) score across all candidates (i.e., $S_T = |RRT_{predicted} - RRT_{measured}|/(S_{T,max} - S_{T,med})$).

In addition, we calculated the MS/MS scores (S_M) for all possible MeO-PCB candidates. We used the similarity coefficient ($\cos\theta$) between the measured and the predicted MS/MS data as an initial score to rank the MeO-PCB candidates. The cos θ assesses the differences between two multivariable vectors, for example, the MS/MS profiles of MeO-PCBs, with a value of zero for completely different vectors and a value of 1 for identical vectors.[15] The initial score was then normalized with the maximal ($S_{M,max}$) and median ($S_{M,med}$) score across all candidates (i.e., $S_M = \cos\theta/(S_{M,max} - S_{M,med})$).

Subsequently, a weighted rank score (*S*) of a candidate was calculated. We initially assessed the true rates (*TR*) with which S_T or S_M ranked the true positive as the top 1 candidate. Briefly, a whole set of MeO-PCBs with one to three chlorines (n=295) were sampled, and the values of RRTs and MS/MS profiles were predicted with the models developed (i.e., MLR model for RRT prediction and RFR model for MS/MS prediction). For di-MeO-PCBs, only compounds with methoxy groups ortho or para to each other were sampled because only PCB catechol and hydroquinone metabolites are formed in metabolism studies.[9, 16, 17] The S_T and S_M values of representative, available mono- to tri-chlorinated MeO-PCBs were ranked, and the true rates were studied. The true rates of S_T rankings (TR_T) was 67 % (n=52) and 19 % (n=51) for MeO-PCB congener and homologs, respectively, while the true rates of S_M rankings (TR_M) were 73 % (n=42, coeluting compounds were removed) and 38 % (n=41, coeluting compounds were

removed) for MeO-PCB congener and homologs, respectively. The weights of S_T (W_T) and S_M (W_M) were calculated as $W_T = TR_T/(TR_T + TR_M)$ and $W_M = 1 - W_T$, respectively. These weights were used to calculate the weighted rank score of a candidate structure as $S = S_T W_T + S_M W_M$. The weighted rank scores of all candidates were divided by the maximal score to receive scores that are scaled from 0 to 1.

Animal experiments. All procedures involving animals were conducted following the NIH Guide for the Care and Use of Laboratory Animals and were approved by the Institutional Animal Care and Use Committee (IACUC) of the University of Arizona or the University of California, Davis.

Sample collection from mice exposed to PCB 95. Adult male or female C57BL/6 mice were exposed to a single oral dose of racemic PCB 95 (1.0 mg/kg) in stripped corn oil (10 ml/kg; lot# A0395699; cat# 801-03-7; Fisher Scientific, Waltham, MA, USA) via oral gavage. PCB95 was synthesized and authenticated as described previously.[18] Control animals received corn oil alone. Animals were euthanized 24 h after the PCB 95 administration; various tissues were dissected and stored for another study. Feces from dissected distal colon and rectum were collected, stored at -80 ^{o}C, and shipped on dry ice to the University of Iowa for the analysis of hydroxylated PCB 95 metabolites.

Sample collection from mice exposed to MARBLES PCB mixture. The liver sample from a male mouse exposed via the maternal diet to the MARBLES PCB mixture was generated at the University of California, Davis, as part of an overall study designed to assess the effects of developmental exposure to the MARBLES PCB mixture on multiple developmental outcomes.[19, 20] Briefly, C57Bl/6J and SVJ129 WT mice were purchased from Jackson Labs (Sacramento, CA) and crossed to generate 75% C57Bl/6J / 25% SVJ129 mice. These mice were used as congenic

wild-type mice in the overall study. All animals were housed in clear plastic shoebox cages containing corn cob bedding and maintained on a 12 h light and dark cycle at 22 ± 2 °C with 40-50% humidity. Feed (Diet 5058, LabDiet, Saint Louis, MO) and water were available *ad libitum*. Two weeks prior to mating, nulliparous and previously unmated dams (>6 weeks of age) were singly housed, and PCB dosing was initiated. Dams were placed with a male overnight for mating. Males and females were separated the next day, and females were checked for the presence of a copulatory plug, which was considered gestational day 0. After mating, dams were housed singly prior to parturition and with their pups after parturition. On postnatal day 2, pups were culled or cross-fostered to ensure all litters consisted of 4-8 pups.

The MARBLES PCB mixture was prepared to mimic the PCB congener profile of the twelve most prevalent PCB congeners detected in the serum of pregnant women enrolled in the MARBLES human epidemiological cohort.[18, 21] These women are at increased risk for having a child with a neurodevelopmental disorder.[22] The PCB congeners included in the MARBLES PCB mixture and their final total percentage in the mixture was as follows: PCB 28 (48.2%), PCB 11 (24.3%), PCB 118 (4.9%), PCB 101 (4.5%), PCB 52 (4.5%), PCB 153 (3.1%), PCB 180 (2.8%), PCB 149 (2.1%), PCB 138 (1.7%), PCB 84 (1.5%), PCB 135 (1.3%) and PCB 95 (1.2%). The MARBLES PCB mix was solubilized in peanut oil and homogeneously mixed into peanut butter, and dams were fed with the MARBLES PCB mix in peanut butter at a dose of 6 mg/kg body weight/day daily until pups were weaned at postnatal day 21. At each daily dosing, dams were monitored to ensure complete ingestion of each dose of peanut butter. Some pups were euthanized at postnatal day 21 (PND 21) for chemical and biochemical analyses. The liver from one PND 21 male mouse was randomly selected for analysis of OH-PCB 28 metabolites.

Livers from several other PND 21 pups from a dam exposed to peanut butter without PCBs were used as controls.

Extraction of the hydroxylated PCBs from samples collected in animal studies. *Analyzing hydroxylated PCB 95 metabolites in the feces of mice exposed to PCB 95.* A feces sample was collected from a male mouse orally exposed to PCB 95 for 24 h and extracted following a published procedure.[23-25] Feces samples from a PCB 95 exposed mouse and a control mouse were incubated with sulfatase (type H-2 from *Helix pomatia*, Sigma-Aldrich, Burlington, MA, USA) to deconjugate PCB sulfate and PCB glucuronide derivatives. Briefly, feces samples (50 mg) were homogenized in 3 mL of 0.2 M sodium acetate buffer (pH 5) and spiked with 4-PCB 52 sulfate (sulfuric acid mono-(2,2′,5,5′-tetrachlorobiphenyl-4-yl) ester, ammonium salt;[6] 100 ng in DMSO). Samples were incubated with 50 µL sulfatase for 16 hours at 37 °C in a shaking water bath. After quenching with 1 mL of 6 M HCl, the samples were spiked with 4′-159 (50 ng) and PCB 117 (50 ng) as surrogate recovery standards, followed by adding 5 mL of 2-propanol and 5 mL of hexane-MTBE mixture (1:1, v/v). The samples were inverted for 5 min and centrifuged at 1,690 g for 5 minutes to facilitate the phase separation. The organic phase was transferred to a new tube, and the aqueous phase was reextracted with 3 mL of hexane. The combined organic phase was washed with 3 mL of 1% KCl aqueous solution, and the resulted organic phase was evaporated to almost dryness under a gentle flow of nitrogen. Then, 0.5 mL of hexane, 5 drops of methanol, and 0.5 mL of diazomethane (about 5 mmol) in diethyl ether were added to derivatize the OH-PCBs to methoxylated derivatives (MeO-PCBs). Samples were kept at 4 °C overnight and excessive diazomethane was evaporated under a gentle flow of nitrogen. The extract was redissolved in 1 mL of hexane and passed through a glass SPE cartridge containing 2 g of acidified silica gel (silica gel : H_2SO_4, 2:1, w/w) with 0.2 of activated silica gel

on the bottom. The analyte was eluted with 14 mL of dichloromethane and the eluent was concentrated to almost dryness with a gentle flow of nitrogen. The residue was redissolved in 4 mL of hexane and the hexane solution was treated with 4 mL of concentrated sulfuric acid for further lipid removal. The organic phase was collected and concentrated to almost dryness under a gentle flow of nitrogen and the sample was spiked with internal standard (50 ng of PCB 204) before GC-MS/MS analysis.

Analyzing hydroxylated PCB 28 metabolites in the liver of mice exposed to the MARBLES PCB mixture. A liver sample and a corresponding control were collected on postnatal day 21 from a male mouse exposed throughout gestation and lactation to the MARBLES PCB mixture via the maternal diet. The liver tissue (~0.3 g) was spiked with 4′-159 (50 ng) and PCB 117 (50 ng) as surrogate recovery standards. After homogenization in in 2-propanol (3 mL) and 1 mL of diethyl ether, the supernatant was transferred to a new tube, the pellet was re-extracted after addition of 2-propanol (1 mL) with hexane : diethyl ether (9:1 v/v, 2.5 mL). The combined extract was washed with phosphoric acid (0.1 M solution in 0.9% aqueous sodium chloride, 5mL), concentrated, and derivatized with diazomethane (0.5 mL) at 4 ^{o}C overnight. The extract was cleaned up using an acidified silica gel column, followed by sulfuric acid treatment, before GC-MS/MS analysis using the MRM method described above. The sample extraction and analysis procedures were the same as described above, except that no sulfuric acid treatment step was employed.

Table S1. List of methoxylated PCBs (MeO-PCBs) used for model training and external testing and their abbreviations and SMILES structures.[a]

Abbreviation	MeO pattern	Cl pattern	SMILES Structures
2-3	2	4	ClC(C=C1OC)=CC=C1C2=CC=CC=C2
2'-3	2'	4	ClC(C=C1)=CC=C1C2=C(OC)C=CC=C2
3- 3	3	4	ClC(C(OC)=C1)=CC=C1C2=CC=CC=C2
2-11	2	3,3'	ClC1=C(OC)C(C2=CC(Cl)=CC=C2)=CC=C1
4,5-1	4,5	2	ClC1=CC(OC)=C(OC)C=C1C2=CC=CC=C2
2',3'-3	2',3'	4	ClC(C=C1)=CC=C1C2=C(OC)C(OC)=CC=C2
6-11	6	3,3'	ClC1=CC=C(OC)C(C2=CC=CC(Cl)=C2)=C1
2',3'-5	2',3'	2,3	ClC1=C(Cl)C(C2=C(OC)C(OC)=CC=C2)=CC=C1
4'-5	4'	2,3	ClC1=C(Cl)C(C2=CC=C(OC)C=C2)=CC=C1
2'-28	2'	2,4,4'	ClC1=CC(Cl)=CC=C1C2=C(OC)C=C(Cl)C=C2
2',5'-8	2',5'	2,4'	ClC1=CC=CC=C1C2=C(OC)C=C(Cl)C(OC)=C2
4,5-4	4,5	2,2'	ClC1=CC(OC)=C(OC)C=C1C2=C(Cl)C=CC=C2
5-11	5	3,3'	ClC1=CC(OC)=CC(C2=CC=CC(Cl)=C2)=C1
3-50	3	2,2',4,6	ClC(C(OC)=C1Cl)=CC(Cl)=C1C2=C(Cl)C=CC=C2
2',5'-5	2',5'	2,3	ClC1=C(Cl)C=CC=C1C2=C(OC)C=CC(OC)=C2
3',4'-3	3',4'	4	ClC(C=C1)=CC=C1C2=CC=C(OC)C(OC)=C2
5,6-11	5,6	3,3'	ClC1=CC(OC)=C(OC)C(C2=CC=CC(Cl)=C2)=C1
2,5-11	2,5	3,3'	ClC1=CC(OC)=CC(C2=CC=CC(Cl)=C2)=C1OC
4-11	4	3,3'	ClC1=C(OC)C=CC(C2=CC=CC(Cl)=C2)=C1
3-28	3	2,4,4'	ClC1=C(OC)C(Cl)=C(C2=CC=C(Cl)C=C2)C=C1
2',3'-12	2',3'	3,4	ClC(C(Cl)=C1)=CC=C1C2=C(OC)C(OC)=CC=C2
2',3'-9	2',3'	2,5	ClC1=CC(C2=C(OC)C(OC)=CC=C2)=C(Cl)C=C1
4'-12	4'	3,4	ClC1=CC(C2=CC=C(OC)C=C2)=CC=C1Cl
4'-35	4'	3,3',4	ClC1=CC(C2=CC(Cl)=C(Cl)C=C2)=CC=C1OC
2',5'-14	2',5'	3,5	ClC1=CC(C2=CC(OC)=CC=C2OC)=CC(Cl)=C1
3'-28	3'	2,4,4'	ClC1=CC(Cl)=C(C2=CC=C(Cl)C(OC)=C2)C=C1
5-28	5	2,4,4'	ClC1=CC(Cl)=C(C2=CC=C(Cl)C=C2)C=C1OC
3',4'-5	3',4'	2,3	ClC1=C(Cl)C=CC=C1C2=CC=C(OC)C(OC)=C2
4,5-8	4,5	2,4'	ClC1=CC(OC)=C(OC)C=C1C2=CC=C(Cl)C=C2
2,5-15	2,5	4,4'	ClC(C=C1)=CC=C1C2=CC(OC)=C(Cl)C=C2OC
4'-29	4'	2,4,5	ClC1=C(Cl)C=C(C2=CC=C(OC)C=C2)C(Cl)=C1
4-36	4	3,3',5	ClC1=C(OC)C(Cl)=CC(C2=CC=CC(Cl)=C2)=C1
4'-25	4'	2,3'4	ClC1=CC(Cl)=C(C2=CC=C(OC)C(Cl)=C2)C=C1
4,5-8	4,5	2,4'	ClC1=C(OC)C(OC)=CC(C2=CC=CC(Cl)=C2)=C1
2',5'-31	2',5'	2,4',5	ClC(C(OC)=C1)=CC(OC)=C1C2=C(Cl)C=CC(Cl)=C2

[a] Rows with light blue background contain MeO-PCBs used for external model testing.

Table S1 (continued). List of methoxylated PCBs (MeO-PCBs) used for model training and external testing and their abbreviations and SMILES structures.[a]

Abbreviation	MeO pattern	Cl pattern	SMILES Structures
3-103	3	2,2',4,5',6	ClC1=CC(C2=C(Cl)C(OC)=C(Cl)C=C2Cl)=C(Cl)C=C1
3',4'-14	3',4'	3,5	ClC1=CC(C2=CC(OC)=C(OC)C=C2)=CC(Cl)=C1
3-100	3	2,2',4,4',6	ClC1=C(C2=C(Cl)C=C(Cl)C=C2)C(Cl)=CC(Cl)=C1OC
4'-68	4'	2,3',4,5'	ClC1=C(C2=CC(Cl)=C(OC)C(Cl)=C2)C=CC(Cl)=C1
3'-98	3'	2,2',3,4',6'	ClC1=C(C2=C(Cl)C=C(Cl)C(OC)=C2Cl)C=CC=C1Cl
3',4'-12	3',4'	3,4	ClC1=CC=C(C2=CC(OC)=C(OC)C=C2)C=C1Cl
3'-150	3'	2,2',3,4',6,6'	ClC1=C(C2=C(Cl)C=C(Cl)C(OC)=C2Cl)C(Cl)=CC=C1Cl
4'-95	4'	2,2',3,5',6	ClC1=C(C2=CC(Cl)=C(OC)C=C2Cl)C(Cl)=CC=C1Cl
5-91	5	2,2',3,4',6	ClC1=C(C2=CC=C(Cl)C=C2Cl)C(Cl)=C(OC)C=C1Cl
4,5-95	4,5	2,2',3,5',6	ClC1=C(C2=CC(Cl)=CC=C2Cl)C(Cl)=C(OC)C(OC)=C1Cl
4-91	4	2,2',3,4',6	ClC1=C(C2=CC=C(Cl)C=C2Cl)C(Cl)=CC(OC)=C1Cl
4,5-91	4,5	2,2',3,4',6	ClC1=C(C2=CC=C(Cl)C=C2Cl)C(Cl)=C(OC)C(OC)=C1Cl
4,5-136	4,5	2,2',3,3',6,6'	ClC1=C(C2=C(Cl)C=CC(Cl)=C2Cl)C(Cl)=C(OC)C(OC)=C1Cl
4-136	4	2,2',3,3',6,6'	ClC1=C(C2=C(Cl)C=CC(Cl)=C2Cl)C(Cl)=CC(OC)=C1Cl
3'-140	3'	2,2',3,4,4',6'	ClC1=C(C2=C(Cl)C=C(Cl)C(OC)=C2Cl)C=CC(Cl)=C1Cl
4,4'-52	4,4'	2,2',5,5'	ClC1=C(C2=CC(Cl)=C(OC)C=C2Cl)C=C(Cl)C(OC)=C1
4,5-132	4,5	2,2',3,3',4,6'	ClC1=C(C2=C(Cl)C=C(Cl)C(Cl)=C2Cl)C=C(OC)C(OC)=C1Cl
2-2	2	3	ClC1=CC=CC(C2=CC=CC=C2)=C1OC
2'-2	2'	3	ClC1=CC=CC(C2=CC=CC=C2OC)=C1
6-2	6	3	ClC1=CC=C(OC)C(C2=CC=CC=C2)=C1
4-1	4	2	ClC1=CC(OC)=CC=C1C2=CC=CC=C2
5-2	5	3	ClC1=CC(OC)=CC(C2=CC=CC=C2)=C1
3'-2	3'	3	ClC1=CC=CC(C2=CC=CC(OC)=C2)=C1
2'-5	2'	2,3	ClC1=C(C=CC=C1C2=C(C=CC=C2)OC)Cl
4-2	4	3	ClC1=C(OC)C=CC(C2=CC=CC=C2)=C1
4'-2	4'	3	ClC1=CC=CC(C2=CC=C(OC)C=C2)=C1
4'-3	4'	4	ClC1=CC=C(C2=CC=C(C=C2)OC)C=C1
2'-30	2'	2,4,6	ClC1=CC(Cl)=CC(Cl)=C1C2=CC=CC=C2OC
3'-9	3'	2,5	ClC1=CC(C2=CC(OC)=CC=C2)=C(Cl)C=C1
2'-12	2'	3,4	ClC(C(Cl)=C1)=CC=C1C2=C(OC)C=CC=C2
4-14	4	3,5	ClC1=CC(C2=CC=CC=C2)=CC(Cl)=C1OC
4'-9	4'	2,5	ClC1=CC(C2=CC=C(OC)C=C2)=C(Cl)C=C1
3'-30	3'	2,4,6	ClC1=CC(Cl)=CC(Cl)=C1C2=CC=CC(OC)=C2

[a] Rows with light blue background contain MeO-PCBs used for external model testing.

Table S1 (continued). List of methoxylated PCBs (MeO-PCBs) used for model training and external testing and their abbreviations and SMILES structures.[a]

Abbreviation	MeO pattern	Cl pattern	SMILES Structures
6'-26	6'	2,3',5	ClC1=C(C2=C(OC)C=CC(Cl)=C2)C=C(Cl)C=C1
4'-18	4'	2,2',5	ClC1=C(C2=CC=C(OC)C=C2Cl)C=C(Cl)C=C1
4'-30	4'	2,4,6	ClC1=CC(Cl)=CC(Cl)=C1C2=CC=C(OC)C=C2
3-54	3	2,2',6,6'	ClC1=C(C2=C(Cl)C=CC=C2Cl)C(Cl)=CC=C1OC
6'-69	6'	2,3',4,6	ClC1=C(C2=C(OC)C=CC(Cl)=C2)C(Cl)=CC(Cl)=C1
2'-65	2'	2,3,5,6	ClC1=C(C2=CC=CC=C2OC)C(Cl)=C(Cl)C=C1Cl
4'-26	4'	2,3',5	ClC1=C(C=C(C=C1)Cl)C2=CC=C(OC)C(Cl)=C2
4-65	4	2,3,5,6	ClC1=C(C2=CC=CC=C2)C(Cl)=C(Cl)C(OC)=C1Cl
3'-65	3'	2,3,5,6	ClC1=C(C2=CC=CC(OC)=C2)C(Cl)=C(Cl)C=C1Cl
2'-61	2'	2,3,4,5	ClC1=C(C2=CC=CC=C2OC)C=C(Cl)C(Cl)=C1Cl
6'-101	6'	2,2',4,5,5'	ClC1=C(C2=C(OC)C(Cl)=CC=C2Cl)C=C(Cl)C(Cl)=C1
4'-72	4'	2,3',5,5'	ClC1=C(C2=CC(Cl)=C(OC)C(Cl)=C2)C=C(Cl)C=C1
4'-69	4'	2,3',4,6	ClC1=C(C2=CC=C(OC)C(Cl)=C2)C(Cl)=CC(Cl)=C1
4'-65	4'	2,3,5,6	ClC1=C(C2=CC=C(OC)C=C2)C(Cl)=C(Cl)C=C1Cl
6'-83	6'	2,2',3,3',5	ClC1=C(C2=C(OC)C=CC(Cl)=C2Cl)C=C(Cl)C=C1Cl
3'-61	3'	2,3,4,5	ClC1=C(C2=CC=CC(OC)=C2)C=C(Cl)C(Cl)=C1Cl
4'-93	4'	2,2',3,5,6	ClC1=C(C2=CC=C(OC)C=C2Cl)C(Cl)=C(Cl)C=C1Cl
4'-61	4'	2,3,4,5	ClC1=C(C2=CC=C(OC)C=C2)C=C(Cl)C(Cl)=C1Cl
4'-79	4'	3,3',4,5'	ClC1=C(Cl)C=CC(C2=CC(Cl)=C(OC)C(Cl)=C2)=C1
4'-101	4'	2,2',4,5,5'	ClC1=C(C2=CC(Cl)=C(OC)C=C2Cl)C=C(Cl)C(Cl)=C1
2'-106	2'	2,3,3',4,5	ClC1=C(Cl)C(Cl)=CC(C2=CC=CC(Cl)=C2OC)=C1Cl
2'-114	2'	2,3,4,4',5	ClC1=C(Cl)C(Cl)=CC(C2=CC=C(Cl)C=C2OC)=C1Cl
3,3'-155	3,3'	2,2',4,4',6,6'	ClC1=C(OC)C(Cl)=C(C2=C(Cl)C=C(Cl)C(OC)=C2Cl)C(Cl)=C1
4'-120	4'	2,3',4,5,5'	ClC1=CC(Cl)=C(C2=CC(Cl)=C(OC)C(Cl)=C2)C=C1Cl
4-134	4	2,2',3,3',5,6	ClC1=C(C2=CC=CC(Cl)=C2Cl)C(Cl)=C(Cl)C(OC)=C1Cl
4'-86	4'	2,2',3,4,5	ClC1=C(Cl)C(Cl)=C(C2=CC=C(OC)C=C2Cl)C=C1Cl
4-97	4	2,2',3,4',5'	ClC1=C(C2=CC(Cl)=C(Cl)C=C2Cl)C=CC(OC)=C1Cl
4'-108	4'	2,3,3',4,5'	ClC1=C(Cl)C(Cl)=C(C2=CC(Cl)=C(OC)C(Cl)=C2)C=C1
3-118	3	2,3',4,4',5	ClC1=C(OC)C(Cl)=C(C2=CC=C(Cl)C(Cl)=C2)C=C1Cl
4-107	4	2,3,3',4',5	ClC1=C(C2=CC=C(Cl)C(Cl)=C2)C=C(Cl)C(OC)=C1Cl
3'-184	3'	2,2',3,4,4',6,6'	ClC1=C(Cl)C(Cl)=C(C2=C(Cl)C=C(Cl)C(OC)=C2Cl)C(Cl)=C1
4-146	4	2,2',3,4',5,5'	ClC1=C(C2=CC(Cl)=C(Cl)C=C2Cl)C=C(Cl)C(OC)=C1Cl
3'-138	3'	2,2',3,4,4',5'	ClC1=C(Cl)C(Cl)=C(C2=CC(Cl)=C(Cl)C(OC)=C2Cl)C=C1
4'-130	4'	2,2',3,3',4,5'	ClC1=C(Cl)C(Cl)=C(C2=CC(Cl)=C(OC)C(Cl)=C2Cl)C=C1

[a] Rows with light blue background contain MeO-PCBs used for external model testing.

Table S1 (continued). List of methoxylated PCBs (MeO-PCBs) used for model training and external testing and their abbreviations and SMILES structures.[a]

Abbreviation	MeO pattern	Cl pattern	SMILES Structures
4'-127	4'	3,3',4,5,5'	ClC1=C(Cl)C=C(C2=CC(Cl)=C(OC)C(Cl)=C2)C=C1Cl
4-178	4	2,2',3,3',5,5',6	ClC1=C(OC)C(Cl)=C(Cl)C(C2=CC(Cl)=CC(Cl)=C2Cl)=C1Cl
4-163	4	2,3,3',4',5,6	ClC1=C(OC)C(Cl)=C(Cl)C(C2=CC=C(Cl)C(Cl)=C2)=C1Cl
3'-182	3'	2,2',3,4,4',5,6'	ClC1=C(Cl)C(Cl)=C(C2=C(Cl)C=C(Cl)C(OC)=C2Cl)C=C1Cl
3'-183	3'	2,2',3,4,4',5',6	ClC1=C(Cl)C(Cl)=C(C2=CC(Cl)=C(Cl)C(OC)=C2Cl)C(Cl)=C1
5-183	5	2,2',3,4,4',5',6	ClC1=C(Cl)C(Cl)=C(C2=CC(Cl)=C(Cl)C=C2Cl)C(Cl)=C1OC
4-187	4	2,2',3,4',5,5',6	ClC1=C(OC)C(Cl)=C(Cl)C(C2=CC(Cl)=C(Cl)C=C2Cl)=C1Cl
5-138	5	2,2',3,4,4',5'	ClC1=C(Cl)C(Cl)=C(C2=CC(Cl)=C(Cl)C=C2Cl)C=C1OC
4-202	4	2,2',3,3',5,5',6,6'	ClC1=C(OC)C(Cl)=C(Cl)C(C2=C(Cl)C(Cl)=CC(Cl)=C2Cl)=C1Cl
4'-177	4'	2,2',3,3',4,5',6'	ClC1=C(Cl)C(Cl)=C(C2=C(Cl)C(Cl)=C(OC)C(Cl)=C2Cl)C=C1
4'-159	4'	2,3',3',4,5,5'	ClC1=C(Cl)C(Cl)=C(C2=CC(Cl)=C(OC)C(Cl)=C2)C=C1Cl
4-162	4	2,3,3',4',5,5'	ClC1=C(OC)C(Cl)=CC(C2=CC(Cl)=C(Cl)C(Cl)=C2)=C1Cl
4'-201	4'	2,2',3,3',4,5',6,6'	ClC1=C(Cl)C(Cl)=C(C2=C(Cl)C(Cl)=C(OC)C(Cl)=C2Cl)C(Cl)=C1
4-193	4	2,3,3',4',5,5',6	ClC1=C(OC)C(Cl)=C(Cl)C(C2=CC(Cl)=C(Cl)C(Cl)=C2)=C1Cl
3'-180	3'	2,2'3,4,4',5,5'	ClC1=C(Cl)C(Cl)=C(C2=CC(Cl)=C(Cl)C(OC)=C2Cl)C=C1Cl
4'-172	4'	2,2',3,3',4,5,5'	ClC1=C(Cl)C(Cl)=C(C2=CC(Cl)=C(OC)C(Cl)=C2Cl)C=C1Cl
4,4'-202	4,4'	2,2',3,3',5,5',6,6'	ClC1=C(OC)C(Cl)=C(Cl)C(C2=C(Cl)C(Cl)=C(OC)C(Cl)=C2Cl)=C1Cl
4'-198	4'	2,2',3,3',4,5,5',6	ClC1=C(Cl)C(Cl)=C(C2=CC(Cl)=C(OC)C(Cl)=C2Cl)C(Cl)=C1Cl
4'-200	4'	2,2',3,3',4,5,6,6'	ClC1=C(Cl)C(Cl)=C(C2=C(Cl)C=C(OC)C(Cl)=C2Cl)C(Cl)=C1Cl
3'-203	3'	2,2',3,4,4',5,5',6	ClC1=C(Cl)C(Cl)=C(C2=CC(Cl)=C(Cl)C(OC)=C2Cl)C(Cl)=C1Cl
4'-199	4'	2,2',3,3',4,5,5',6'	ClC1=C(Cl)C(Cl)=C(C2=C(Cl)C(Cl)=C(OC)C(Cl)=C2Cl)C=C1Cl
4'-208	4'	2,2',3,3',4,5,5',6,6'	ClC1=C(Cl)C(Cl)=C(C2=C(Cl)C(Cl)=C(OC)C(Cl)=C2Cl)C(Cl)=C1Cl

[a] Rows with light blue background contain MeO-PCBs used for external model testing.

Table S2. List of the optimal predictors and their linear coefficients and p-values that were obtained in the multiple linear regression (MLR) model development to predict the relative retention time (RRT) of MeO-PCBs. For more details about the MLR model development, see the Experimental Section.

Predictors	Coefficient (standard deviation)	p-value
WPOL	-2.1 (0.1)	< 0.0001
WTPT.1	5.6 (0.4)	< 0.0001
WTPT.3	3.0 (0.3)	< 0.0001
MDEO.22	-0.7 (0.1)	< 0.0001
SP.2	-23 (2)	< 0.0001
SP.3	-3.5 (0.3)	< 0.0001
VP.3	1.8 (0.1)	< 0.0001
SC.3	-8.6 (0.7)	< 0.0001
VC.3	26 (2)	< 0.0001
VCH.6	137 (10)	< 0.0001

Table S3. List of the optimal predictors and parameters obtained in the random forest regression (RFR) model development to predict MS/MS data (expressed as relative levels of five MS transitions) of MeO-PCBs. For more details about the RFR model development, see the Experimental Section.

MS transitions	Loss of 15 [CH_3]	Loss of 30 [CH_2O]	Loss of 43 [CH_3+CO]	Loss of 50 [CH_3+Cl]	Loss of 66 [CH_3O+Cl]
Predictors[a]	fragC<br>BCUTc.1l<br>MDEC.13<br>$N_{MeO,para}$<br>BCUTp.1l<br>MDEC.12<br>SP.5<br>VP.5<br>WTPT.4	ATSc3<br>VPC.5<br>SPC.5<br>$N_{MeO,meta}$<br>MDEC.12<br>BCUTc.1l<br>BCUTp.1l<br>MDEC.13<br>WTPT.4	MDEC.13<br>WTPT.1<br>BCUTp.1l<br>MDEC.12<br>topoShape<br>ECCEN<br>nAtomLC<br>$N_{MeO,ortho}$<br>PetitjeanNumber<br>WTPT.4	BCUTp.1l<br>$N_{Cl,meta}$<br>$N_{Cl,para}$<br>ATSc3<br>BCUTc.1l<br>ECCEN<br>nAtomLC<br>$N_{MeO,ortho}$<br>$N_{MeO,para}$<br>WTPT.4	ECCEN<br>SP.2<br>WPATH<br>BCUTp.1l<br>ATSc3<br>ATSc4<br>BCUTc.1l<br>MDEC.12<br>MDEC.13
ntree[b]	200	500	900	800	600
mtry[c]	9	5	5	2	2

[a] $N_{MeO,ortho}$, $N_{MeO,meta}$, $N_{MeO,para}$, $N_{Cl,meta}$ and $N_{Cl,para}$ are substitution pattern-based molecular descriptors of MeO-PCBs, as defined in the Experimental Section. The rest are chemoinformatics-based molecular descriptors that extracted with R package *rcdk*[14] coupled with SMILES structures of MeO-PCBs.

[b] ntree, number of trees to grow.

[c] mtry, number of variables randomly sampled as candidates at each split.

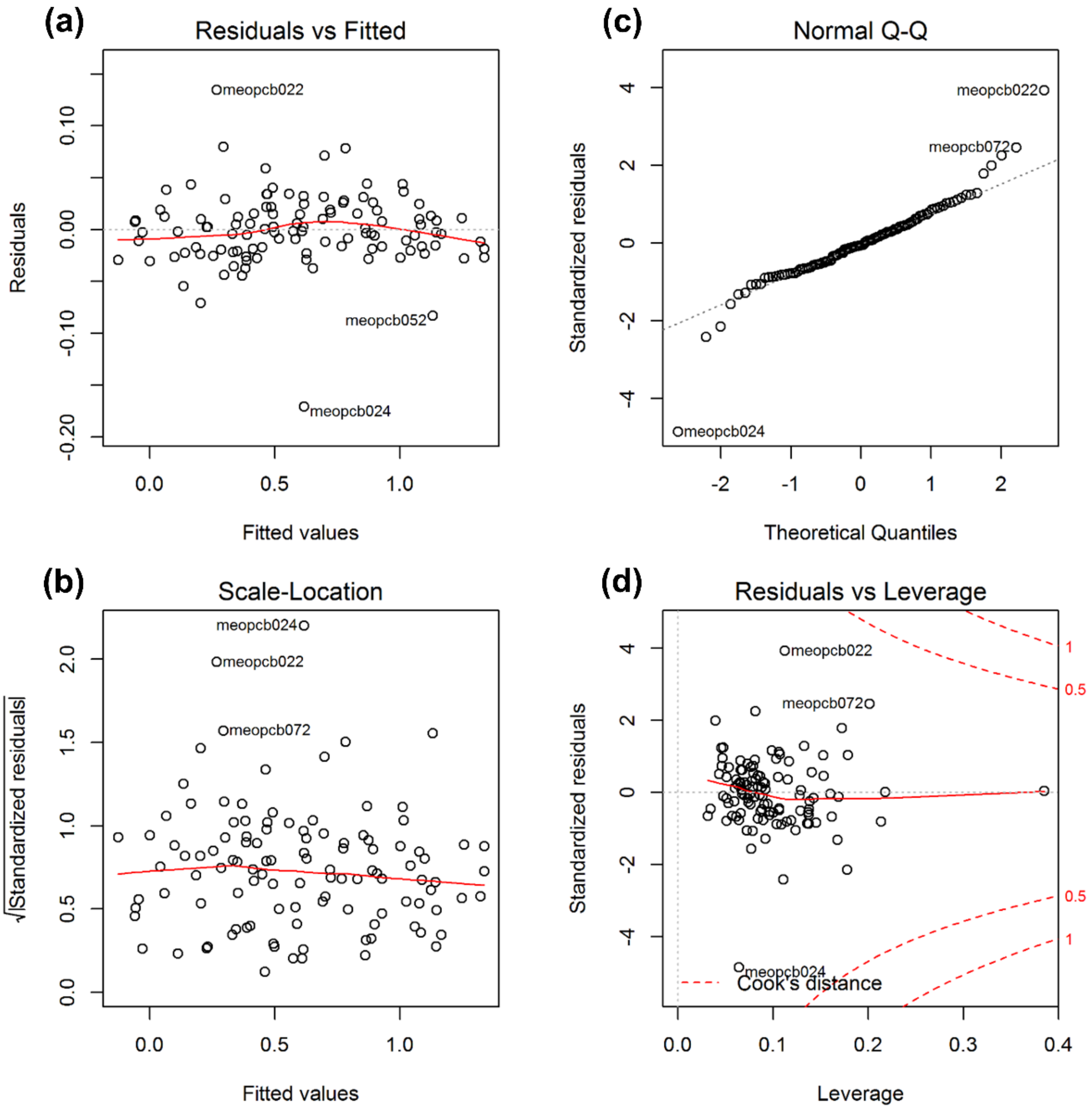

Fig. S1. Diagnostic plots [(a) residuals versus fitted values, (b) scale location, (c) normal Q–Q and (d) residuals versus leverage] for multiple linear regression for predicting relative retention times (RRT) of MeO-PCBs reveal no non-linear relationship between the predictors and the RRTs and support normal data distribution and homogeneity of data variance in the model development.

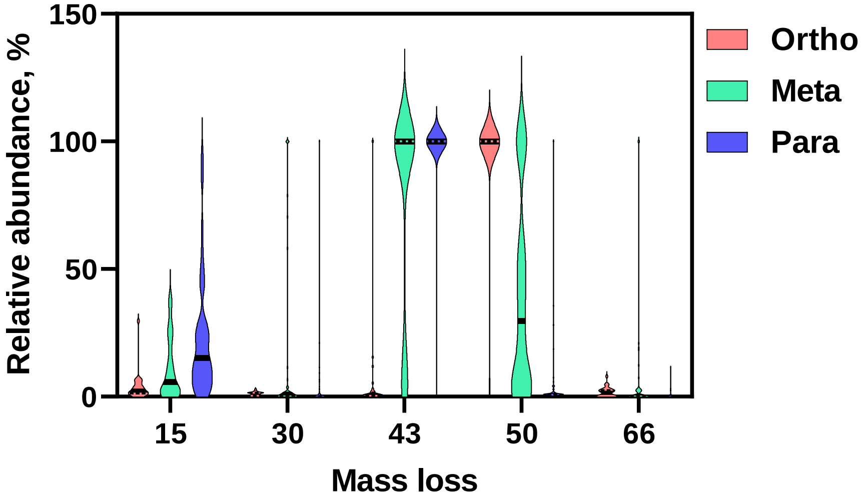

Fig. S2. Five representative MS/MS responses associated with mass losses of 15 [CH_3], 30 [CH_2O], 43 [CH_3+CO], 50 [CH_3+Cl] and 66 [CH_3O+Cl] of MeO-PCBs varied with the substitution positions (ortho-, meta- and para-) of the methoxy group.

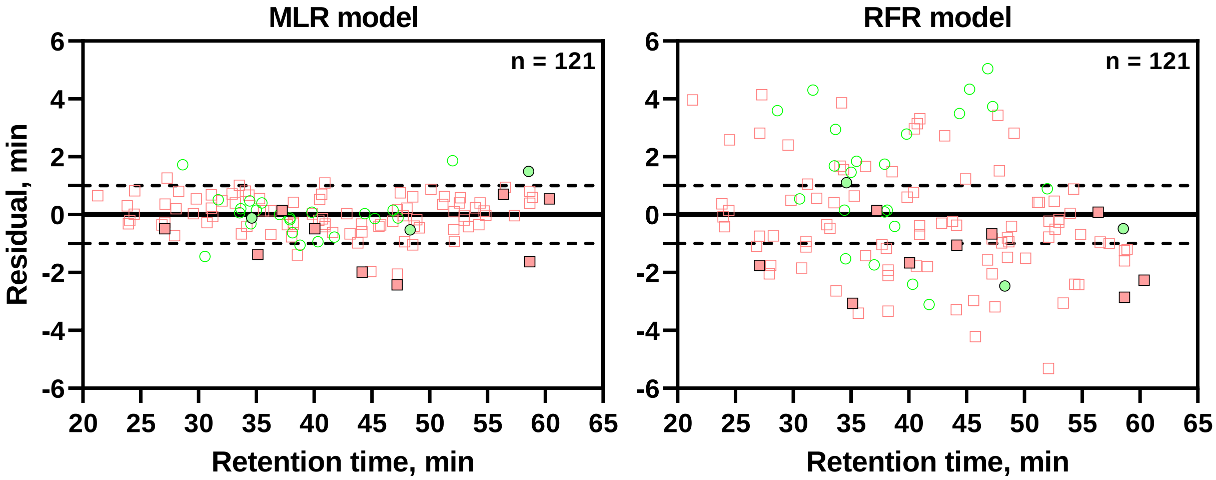

Fig. S3. Plots of absolute differences between measured and predicted RRTs versus experimental retention times revealed randomly distributed residuals in MLR and RFR model predictions. A narrower residual range was observed for the MLR model than the RFR model. For more information about the model development and the experimental determination of RRTs, see the Experimental Section.

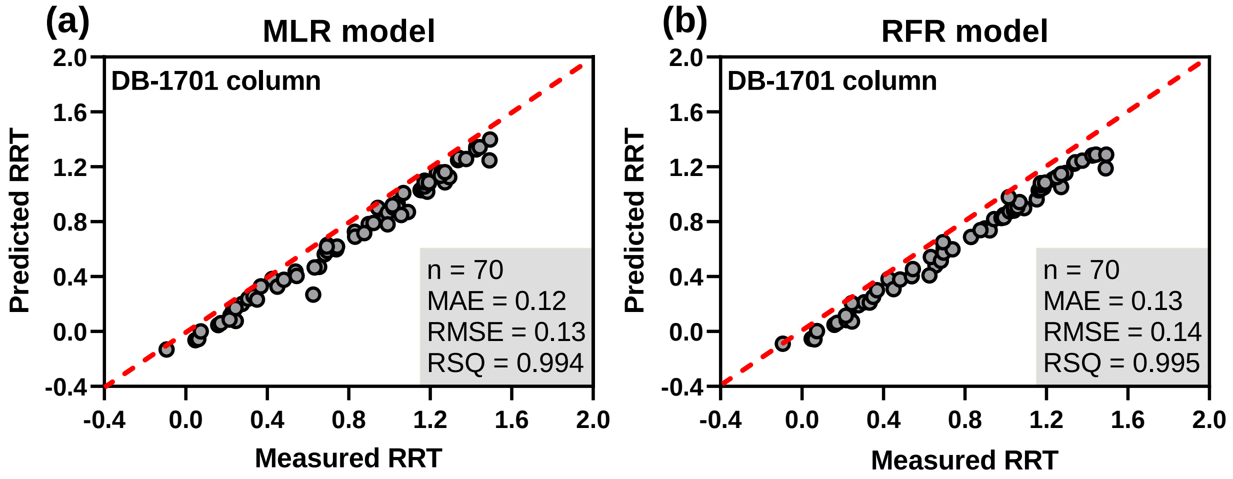

Fig. S4. Both (a) multiple linear regression (MLR) and (b) random forest regression (RFR) models developed with SPB-Octyl column slightly underestimated the relative retention times (RRTs) of MeO-PCBs measured with a DB-1701 column. The RRT data of MeO-PCBs on a DB-1701 column were published previously.[3] For more details regarding the MLR and RFR model development, see the Experimental Section.

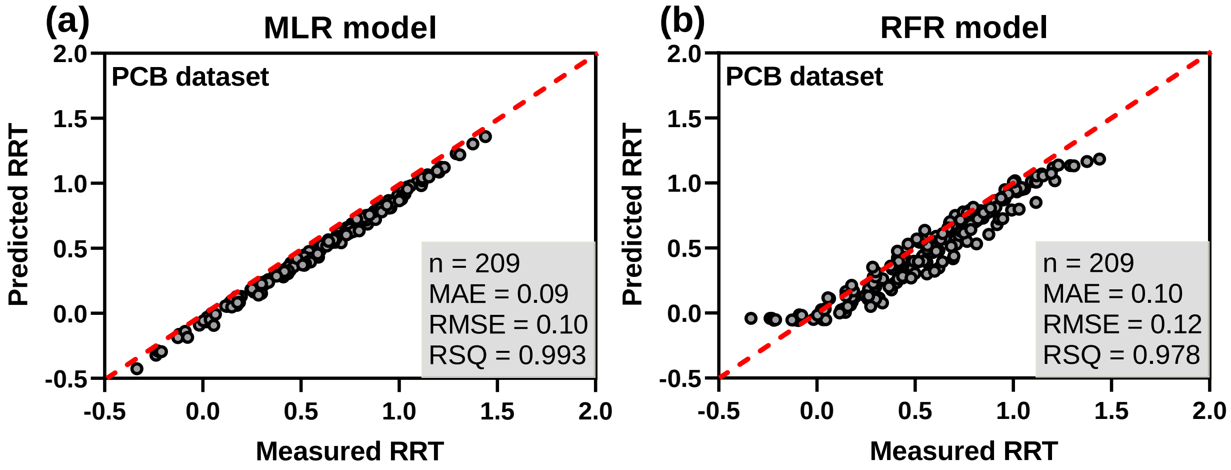

Fig. S5. The (a) multiple linear regression (MLR) model developed for predicting the relative retention times (RRTs) of MeO-PCBs can also provide reasonable estimations of the RRTs of PCBs collected with a SPB-Octyl GC column with a physically different instrument and performed better than (b) the random forest regression (RFR) model in predicting the RRTs of PCBs. The method conditions for measuring the RRTs of PCBs were identical with the MeO-PCBs method, as described in the Experimental Section.

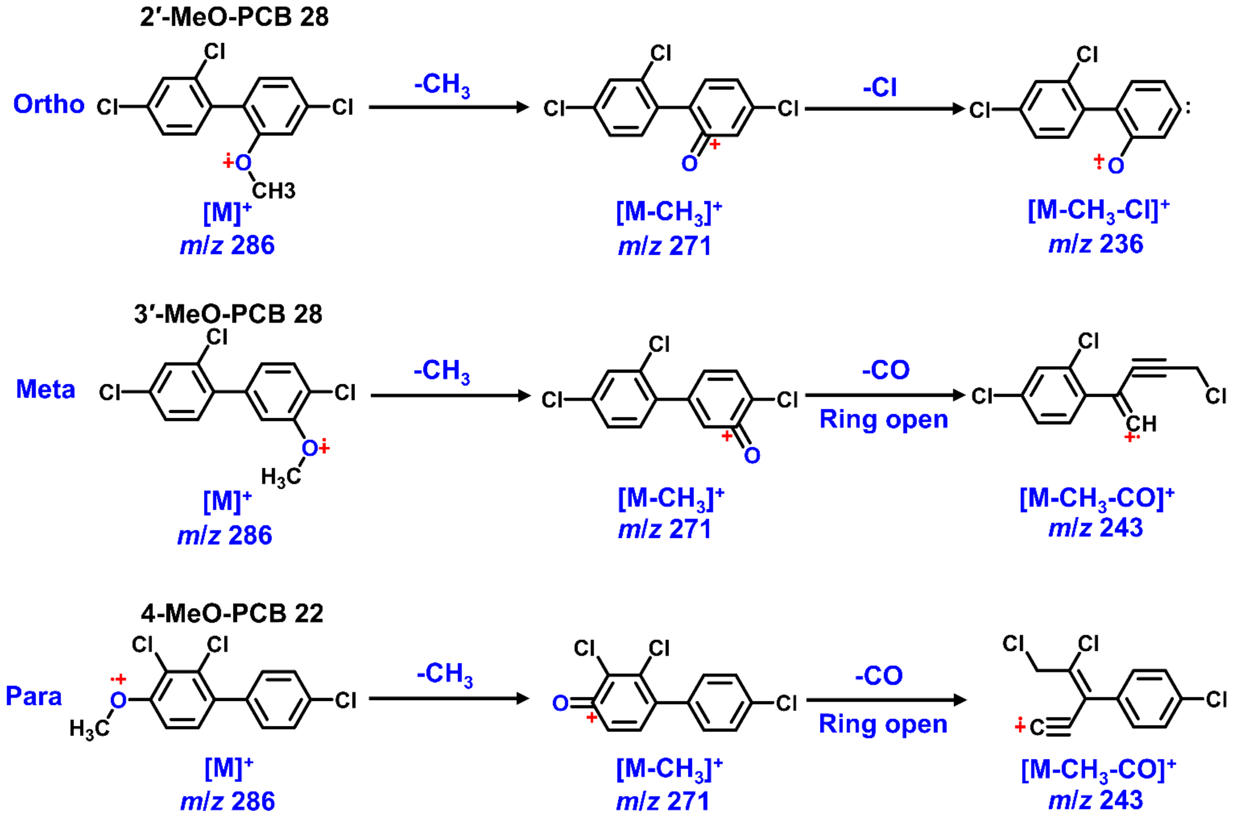

Fig. S6. The predominant fragmentation pathways of MeO-PCBs with different substitution positions (ortho, meta and para) of the methoxy group. MeO-PCB 28 metabolites, including 2′-MeO-PCB 28, 3′-MeO-PCB 28 and 4-MeO-PCB 22 (a 1,2 shift product of PCB 28), were taken as examples. The proposed fragmentation pathways were based on experimentally observed fragment ions of MeO-PCBs.

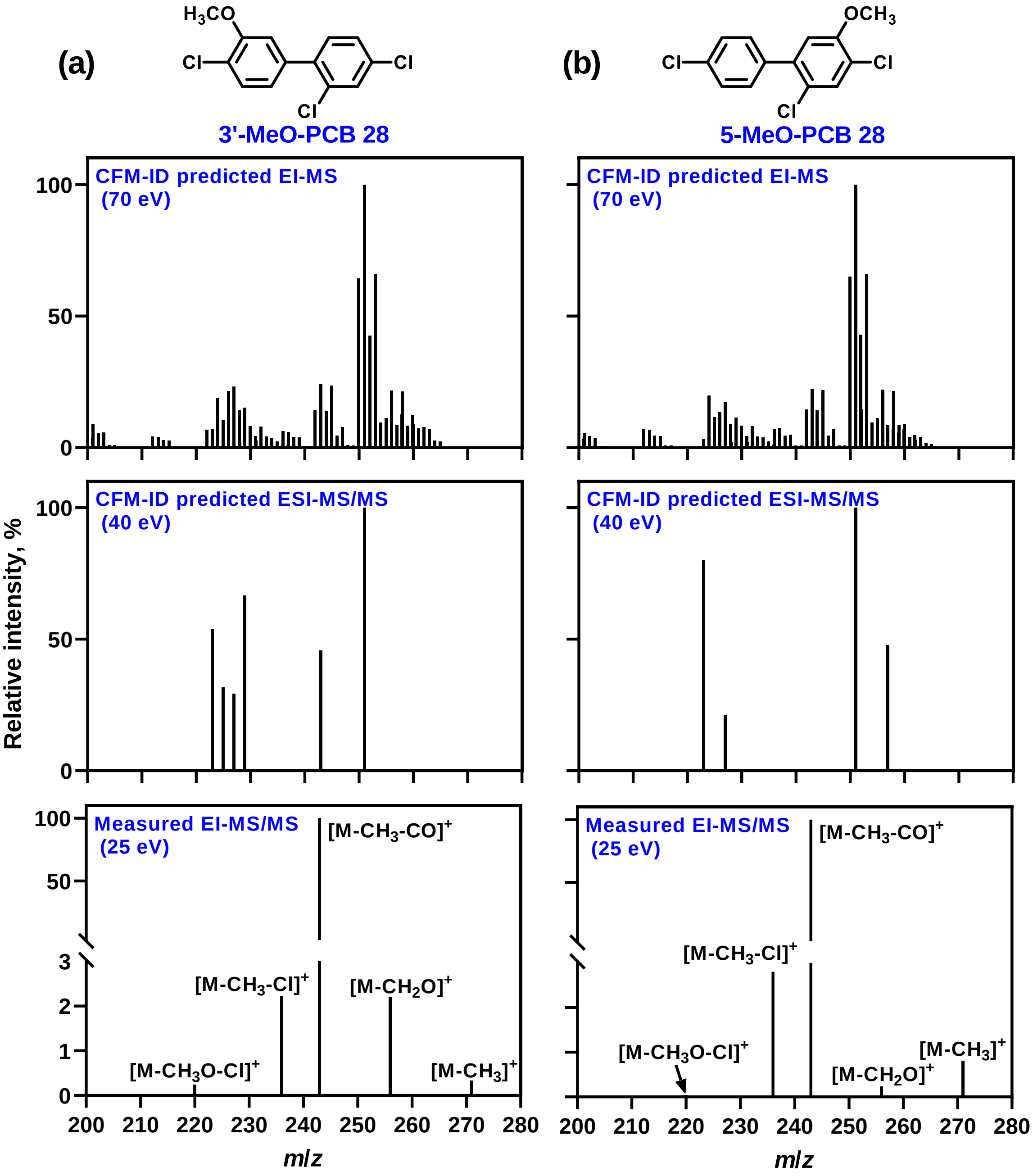

Fig. S7. The comparison between measured and predicted spectra suggests CFM-ID with either EI-MS or ESI-MS/MS modules poorly simulated the fragmentation of MeO-PCBs. 3′-MeO-PCB 28 and 5-MeO-PCB 28 were taken as examples. CFM-ID version 4.0[26] and version 3.0[27] were used for the simulation of ESI-MS/MS and EI-MS spectra of MeO-PCBs, respectively.

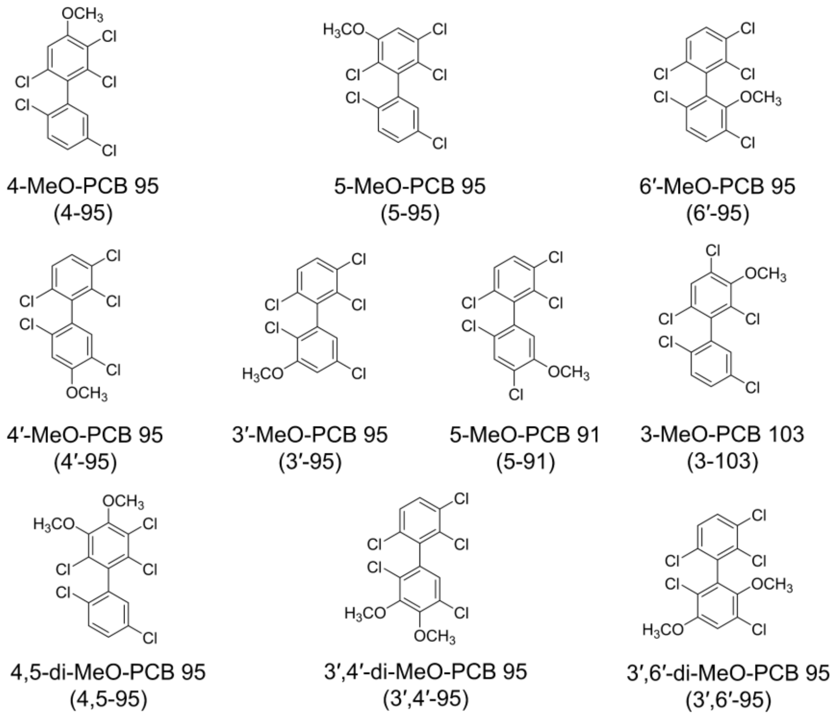

Fig. S8. The abbreviations and their corresponding structures of all possible MeO-PCB 95 metabolites.[28] The abbreviations under the structures correspond to the abbreviations of the MeO-PCB 95 in Fig. 5.

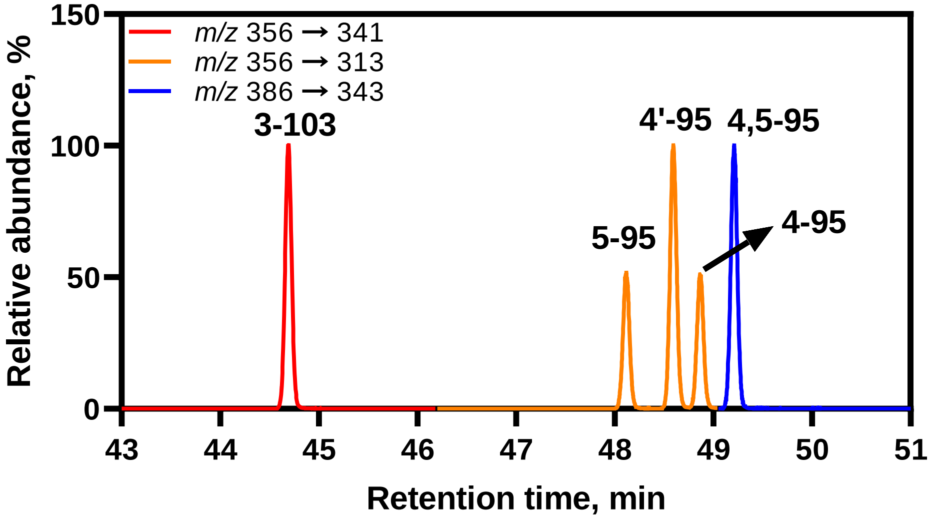

Fig. S9. GC-MS/MS chromatograms of the authentic standards of mono- and di-hydroxylated metabolites of PCB 95 (analyzed as methylated derivatives). The GC-MS/MS was equipped with a SPB-Octyl capillary column. The following temperature program was used for the analysis of PCB 95 and its metabolites: initial temperature of 45 °C, hold for 2 min, 100 °C/min to 75 °C, hold for 5 min, 15 °C/min to 150 °C, hold for 1 min, then 2.5 °C/min to 280 °C, and hold for 5 min. The transfer line temperature was 280 °C.

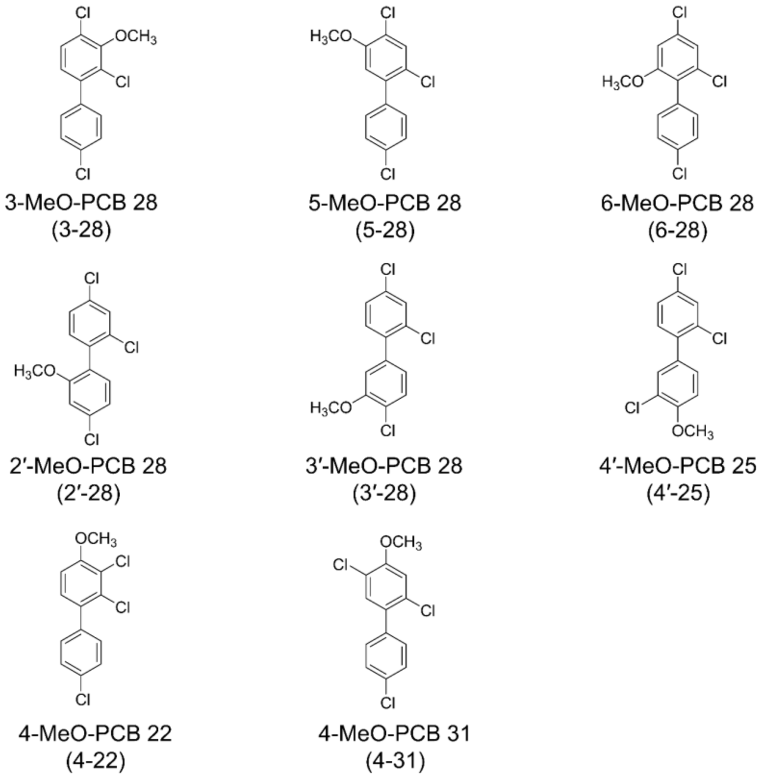

Fig. S10. The abbreviations and their corresponding structures of all possible mono-MeO-PCB 28 metabolites. The abbreviations under the structures correspond to the abbreviations of the MeO-PCB 28 in Fig. 6.

References

1. Lehmler, H. J.; Robertson, L. W. Synthesis of polychlorinated biphenyls (PCBs) using the Suzuki-coupling. *Chemosphere* **2001,** *45*, 137-143.
2. Lehmler, H. J.; Robertson, L. W. Synthesis of hydroxylated PCB metabolites with the Suzuki-coupling. *Chemosphere* **2001,** *45*, 1119-1127.
3. Saktrakulkla, P.; Dhakal, R. C.; Lehmler, H. J.; Hornbuckle, K. C. A semi-target analytical method for quantification of OH-PCBs in environmental samples. *Environ. Sci. Pollut. Res.* **2020,** *27*, 8859-8871.
4. Dhakal, R.; Li, X.; Parkin, S. R.; Lehmler, H.-J. Synthesis of mono-and dimethoxylated polychlorinated biphenyls derivatives starting from fluoroarene derivatives. *Environ. Sci. Pollut. Res.* **2020,** *27*, 8905-8925.
5. Li, X.; Holland, E. B.; Feng, W.; Zheng, J.; Dong, Y.; Pessah, I. N.; Duffel, M. W.; Robertson, L. W.; Lehmler, H. J. Authentication of synthetic environmental contaminants and their (bio)transformation products in toxicology: polychlorinated biphenyls as an example. *Environ. Sci. Pollut. Res.* **2018,** *25*, 16508-16521.
6. Rodriguez, E. A.; Li, X.; Lehmler, H. J.; Robertson, L. W.; Duffel, M. W. Sulfation of Lower Chlorinated Polychlorinated Biphenyls Increases Their Affinity for the Major Drug-Binding Sites of Human Serum Albumin. *Environ. Sci. Technol.* **2016,** *50*, 5320-5327.
7. Zhu, Y. M.; Mapuskar, K. A.; Marek, R. F.; Xu, W. J.; Lehmler, H. J.; Robertson, L. W.; Hornbuckle, K. C.; Spitz, D. R.; Aykin-Burns, N. A New Player in Environmentally Induced Oxidative Stress: Polychlorinated Biphenyl Congener, 3,3-Dichlorobiphenyl (PCB11). *Toxicol. Sci.* **2013,** *136*, 39-50.

8. Zhai, G. S.; Lehmler, H. J.; Schnoor, J. L. New hydroxylated metabolites of 4-monochlorobiphenyl in whole poplar plants. *Chem. Cent. J.* **2011,** *5*, 87.
9. McLean, M. R.; Bauer, U.; Amaro, A. R.; Robertson, L. W. Identification of catechol and hydroquinone metabolites of 4-monochlorobiphenyl. *Chem. Res. Toxicol.* **1996,** *9*, 158-164.
10. Maervoet, J.; Covaci, A.; Schepens, P.; Sandau, C. D.; Letcher, R. J. A reassessment of the nomenclature of polychlorinated biphenyl (PCB) metabolites. *Environ. Health Perspect.* **2004,** *112*, 291-294.
11. Marek, R. F.; Thome, P. S.; Herkert, N. J.; Awad, A. M.; Hornbuckle, K. C. Airborne PCBs and OH-PCBs inside and outside urban and rural US schools. *Environ. Sci. Technol.* **2017,** *51*, 7853-7860.
12. Marek, R. F.; Thorne, P. S.; Wang, K.; DeWall, J.; Hornbuckle, K. C. PCBs and OH-PCBs in serum from children and mothers in urban and rural U.S. communities. *Environ. Sci. Technol.* **2013,** *47*, 9555-9556.
13. Marek, R. F.; Martinez, A.; Hornbuckle, K. C. Discovery of hydroxylated polychlorinated biphenyls (OH-PCBs) in sediment from a lake Michigan waterway and original commercial Aroclors. *Environ. Sci. Technol.* **2013,** *47*, 8204-8210.
14. Rajarshi Guha; Charlop-Powers, Z.; Schymanski, E. rcdk: Interface to the 'CDK' Libraries. https://cran.r-project.org/web/packages/rcdk/index.html (Accessed on Jul 30, 2022),
15. Davis, J. C., Statistics and data analysis in geology. 3rd ed.; John Wiley & Sons: New York, 2002; p 540.
16. Zhang, C.-Y.; Flor, S.; Ruiz, P.; Dhakal, R.; Hu, X.; Teesch, L. M.; Ludewig, G.; Lehmler, H.-J. 3,3'-Dichlorobiphenyl is metabolized to a complex mixture of oxidative metabolites,

including novel methoxylated metabolites, by HepG2 cells. *Environ. Sci. Technol.* **2020,** *54*, 12345-12357.

17. Dhakal, K.; Uwimana, E.; Adamcakova-Dodd, A.; Thorne, P. S.; Lehmler, H. J.; Robertson, L. W. Disposition of phenolic and sulfated metabolites after inhalation exposure to 4-chlorobiphenyl (PCB3) in female rats. *Chem. Res. Toxicol.* **2014,** *27*, 1411-1420.
18. Sethi, S.; Morgan, R. K.; Feng, W.; Lin, Y.; Li, X.; Luna, C.; Koch, M.; Bansal, R.; Duffel, M. W.; Puschner, B.; Zoeller, R. T.; Lehmler, H.-J.; Pessah, I. N.; Lein, P. J. Comparative analyses of the 12 most abundant PCB congeners detected in human maternal serum for activity at the thyroid hormone receptor and ryanodine receptor. *Environ. Sci. Technol.* **2019,** *53*, 3948-3958.
19. Rude, K. M.; Pusceddu, M. M.; Keogh, C. E.; Sladek, J. A.; Rabasa, G.; Miller, E. N.; Sethi, S.; Keil, K. P.; Pessah, I. N.; Lein, P. J.; Gareau, M. G. Developmental exposure to polychlorinated biphenyls (PCBs) in the maternal diet causes host-microbe defects in weanling offspring mice. *Environ. Pollut.* **2019,** *253*, 708-721.
20. Matelski, L.; Keil Stietz, K. P.; Sethi, S.; Taylor, S. L.; Van de Water, J.; Lein, P. J. The influence of sex, genotype, and dose on serum and hippocampal cytokine levels in juvenile mice developmentally exposed to a human-relevant mixture of polychlorinated biphenyls. *Curr. Res. Toxicol.* **2020,** *1*, 85-103.
21. Granillo, L.; Sethi, S.; Keil, K. P.; Lin, Y.; Ozonoff, S.; Iosif, A. M.; Puschner, B.; Schmidt, R. J. Polychlorinated biphenyls influence on autism spectrum disorder risk in the MARBLES cohort. *Environ. Res.* **2019,** *171*, 177-184.
22. Hertz-Picciotto, I.; Schmidt, R. J.; Walker, C. K.; Bennett, D. H.; Oliver, M.; Shedd-Wise, K. M.; LaSalle, J. M.; Giulivi, C.; Puschner, B.; Thomas, J.; Roa, D. L.; Pessah, I. N.; Van

de Water, J.; Tancredi, D. J.; Ozonoff, S. A Prospective Study of Environmental Exposures and Early Biomarkers in Autism Spectrum Disorder: Design, Protocols, and Preliminary Data from the MARBLES Study. *Environ. Health Perspect.* **2018,** *126*, 117004.

23. Kania-Korwel, I.; Duffel, M. W.; Lehmler, H. J. Gas chromatographic analysis with chiral cyclodextrin phases reveals the enantioselective formation of hydroxylated polychlorinated biphenyls by rat liver microsomes. *Environ. Sci. Technol.* **2011,** *45*, 9590-9596.

24. Egusquiza, R. J.; Ambrosio, M. E.; Wang, S. G.; Kay, K. M.; Zhang, C.; Lehmler, H.-J.; Blumberg, B. Evaluating the Role of the Steroid and Xenobiotic Receptor (SXR/PXR) in PCB-153 Metabolism and Protection against Associated Adverse Effects during Perinatal and Chronic Exposure in Mice. *Environ. Health Perspect.* **2020,** *128*, 47011.

25. Wu, X. A.; Pramanik, A.; Duffel, M. W.; Hrycay, E. G.; Bandiera, S. M.; Lehmler, H. J.; Kania-Korwel, I. 2,2 ',3,3 ',6,6 '-Hexachlorobiphenyl (PCB 136) is enantioselectively oxidized to hydroxylated metabolites by rat Liver microsomes. *Chem. Res. Toxicol.* **2011,** *24*, 2249-2257.

26. Wang, F.; Liigand, J.; Tian, S.; Arndt, D.; Greiner, R.; Wishart, D. S. CFM-ID 4.0: More Accurate ESI-MS/MS Spectral Prediction and Compound Identification. *Anal. Chem.* **2021,** *93*, 11692-11700.

27. Djoumbou-Feunang, Y.; Pon, A.; Karu, N.; Zheng, J.; Li, C.; Arndt, D.; Gautam, M.; Allen, F.; Wishart, D. S. CFM-ID 3.0: Significantly Improved ESI-MS/MS Prediction and Compound Identification. *Metabolites* **2019,** *9*, 72.

28. Ma, C. X.; Zhai, G. S.; Wu, H. M.; Kania-Korwel, I.; Lehmler, H. J.; Schnoor, J. L. Identification of a novel hydroxylated metabolite of 2,2 ',3,5 ',6-pentachlorobiphenyl formed in whole poplar plants. *Environ. Sci. Pollut. Res.* **2016,** *23*, 2089-2098.
